# Evaluation of Sort Recovery via *Rmax*

**DOI:** 10.1002/cpz1.986

**Published:** 2024-02-16

**Authors:** Alexis Perez‐Gonzalez, Telma Lopes, Lola Martinez, Claudia Bispo, Rui Gardner, Andy Riddell

**Affiliations:** ^1^ Department of Microbiology and Immunology The University of Melbourne, at The Peter Doherty Institute for Infection and Immunity Parkville Victoria Australia; ^2^ Melbourne Cytometry Platform The University of Melbourne Parkville Victoria Australia; ^3^ Roche Pharma Research and Early Development (pRED) Basel Switzerland; ^4^ Flow Cytometry Core Unit. Spanish National Cancer Research Center (CNIO) Madrid Spain; ^5^ Flow Cytometry Core Facility AbbVie Biotherapeutics Inc. South San Francisco California; ^6^ Flow Cytometry Core Facility Memorial Sloan Kettering Cancer Center New York New York; ^7^ Flow Cytometry Science and Technology Platform The Francis Crick Institute London United Kingdom

**Keywords:** cell sorter performance, cell sorting, cell sorting optimization and troubleshooting, quality control, sort recovery

## Abstract

Cell sorting performance can be evaluated in regard to the purity and recovery of the sorted fractions. The purity provides checks on sample quality, acquisition settings, gating strategy, and the sort decisions made by the instrument, but alone it is not sufficient to evaluate sorting performance. Recovery, defined here as the number of target particles sorted relative to the number of original target particles to be sorted, is a key metric of sort fitness and performance but is often neglected due to difficulties in its measurement. Both purity and recovery require re‐sampling of the sorted fraction, but unlike determining purity, calculating recovery calls for the absolute counting of particles in the sorted fraction that comes with large errors, and may not be feasible for rare populations or precious samples.

Here, we describe a recently developed metric and method for calculating sort recovery called *Rmax*, representing the maximum expected recovery for a particular set of instrument settings. *Rmax* calculation avoids re‐sampling of the total sorted fraction and absolute counting, being instead based on the ratios of target and non‐target populations in the original pre‐sort sample and in the waste stream or center stream catch. The *Rmax* method is ideal to evaluate and troubleshoot the optimum drop‐charge delay of the sorter or any instrument‐related failures that will affect sort performance. It can be used as a daily quality control check but can be particularly useful to assess instrument fitness before single‐cell or rare population sorts. Because the sorted fraction is not perturbed, we can calculate *Rmax* during the sort run. © 2024 The Authors. Current Protocols published by Wiley Periodicals LLC.

**Basic Protocol 1**: Evaluating sorter setup with *Rmax*

**Basic Protocol 2**: Finding the maximum *Rmax*: scanning over the drop charge delay

**Alternate Protocol**: Finding the maximum *Rmax* for cells: scanning over the drop charge delay

**Basic Protocol 3**: Estimating sorted cell number with *Rmax*

## INTRODUCTION

Cell sorting is a vital tool in biological studies. Its principal role is to purify subpopulations of cells or particles from suspensions of complex biological samples, allowing further analysis (Ibrahim & van den Engh, 2007; Vitelli et al., 2021). Typically, cell sorters take monodispersed particles in a sample and accelerate them into a single‐file procession via hydrodynamic forces. The particles then pass one at a time through focused laser beams where parallel measurements of scatter and fluorescence are taken. These measurements allow particles to be classified into sort target and contaminants based on specified criteria or sort logic. Particles satisfying the sort criteria are subsequently deflected by the instrument into a container. The deflection can be mechanical, electrostatic, or a disruptive flow process and happens at a fixed distance from the laser measurements. The timing of the deflection impulse is critical in recovering the sort target.

The expectation is that for every deflection instructed by the electronics, the sort‐classified target particles associated with it will be deflected into the collection container. However, this ideal outcome, representing a full recovery of target particles, is strictly dependent on and challenged by a great variety of factors contributing to the success of the sort process. Issues with sort recovery can derive from a combination of instrument‐intrinsic and ‐extrinsic factors, including sort subsystems malfunction, poor quality of the sample preparation, or inappropriate instrument setup and operation. It is crucial that the original sample is monodispersed, and care should be taken during its preparation to remove excessively large contaminants via filtration and to minimize clumping prior to or while running on a sorter. Large particles and cell clumps will affect the laminar flow stability of the carrier medium and can disrupt the deflection process or, in the worst‐case scenario, block the flow altogether. They also create sort criteria misclassification, contaminating the sort or leading to excessive aborts from the “doublet‐exclusion” circuitry, thereby reducing the recovery of target cells. Additionally, sorters are exquisitely sensitive to vibration and fluidic instabilities, and must be free of dirt and air bubbles along the sheath and sample lines or nozzle and guarantee an accurate definition of the timing from classifying the particles to the physical position where the deflection occurs. Accurate deflection timing is critical for good recovery, as otherwise particle‐free deflections will occur while target particles are lost to waste. It has been shown that small errors in the deflection timing will reduce the recovery but not the purity of the sort target cells (Riddell et al., 2015). Finally, the linear velocity of the analyzed particles must be exclusively dictated by the surrounding sheath flow, and thus expected not to deviate from the predefined delta time between classification and deflection points.

Both purity and recovery are standard metrics to assess cell sorters performance. Although purity is key in informing on the quality of the sorted fractions for downstream processing such as sequencing or cell culture, it is not useful to identify issues with instrument performance affecting sort outcomes. A much better metric of quality control for cell sorters’ performance is recovery, defined as the fraction of the number of target particles collected by sorting, relative to the number of sort‐qualifying particles present in the original sample (Shapiro, 2004, pp. 267). Despite its critical importance, recovery is rarely used in daily evaluations of sort performance. One major reason for this is that particle counting, key in traditional recovery assessments, calls for the destructive testing of the sort collection, reducing cell numbers for downstream processing. In some cases, absolute counting‐based recovery calculations are not practical or possible, particularly during cell sorts for very rare events where the sorted particle number is limited, or during single‐cell sorting into a lysis buffer, ahead of ‐omics applications. Even in situations where it may be possible to do so, absolute counting of suspended particles comes with its own errors and inaccuracies (Dacie & Lewis, 1984; Kirkman‐Brown & Björndahl, 2009; Lapping et al., 1972; Pegg & Antcliff, 1965). In the absence of embedded recovery metrics, instruments report sort efficiency, a recovery surrogate defined as the ratio between sorted target particle counts (based on electronic sort decisions) and the total number of target particles within the sort logic or sort gates. Although this reported metric is used to anticipate the recovery of the instrument while sorting, it is not a direct measurement of instrument performance and sort outcome. In fact, no commercial cell sorter yet has embedded tools to assess and confirm the very thing that they are built to do: to ensure that all reported sort classified particles are successfully deflected into the collection vessel during the sort run.

Maximum recovery or *Rmax* (Riddell et al., 2015) is a nondestructive means to evaluate sorter performance based on the measurement of target and non‐target ratios in the acquired pre‐sort or *original* sample and in the center stream collected during the sort run (for the purposes of this protocol, the collected waste stream is referred to as the *center stream catch* or CSC). As a ratiometric method for recovery, *Rmax* bypasses the need of absolute counting, avoiding the errors associated with traditional recovery calculation procedures. Additionally, *Rmax* allows the assessment of instrument recovery at any given time during a sort, rather than only after completion. *Rmax* can be used as a quality control metric to monitor and troubleshoot factors affecting cell sorter performance before and during a sort, and to evaluate the accuracy of the manufacturer‐recommended method to calculate the sort deflection delays, henceforth referred to as the drop charge delay (DCD).

Usually, 10,000 events from the original sample should be acquired and the number of target and non‐target particles recorded. While the sort is in progress, the CSC is collected and then acquired on a flow cytometer, where, again, the number of target and non‐target particles are recorded. The original and CSC target and non‐target counts are entered into Equation 1 and *Rmax* is calculated.

(1) $$Rmax\;=\;1-\frac{O_{nt}}{O_{t}}\cdot \frac{C_{t}}{C_{nt}}$$

where:

*O_nt_* is the non‐target count in the original sample;

*O_t_* is the target count in the original sample;

*C_t_* is the target count in the CSC;

*C_nt_* is the non‐target count in the CSC.

The protocols outlined here include the evaluation of sorter setup with *Rmax*. Basic Protocol 1 evaluates sorter recovery performance using beads as a “perfect reagent.” This protocol is the foundation of all the other protocols presented here. *Rmax* scanning through different DCD values in the Basic Protocol 2 can be used to find the maximum recovery and to assess the accuracy of the DCD defined during the sorter setup. Alternate Protocol finds the optimum DCD leading to maximum recovery using cells rather than beads. It is useful to maximize recovery for cell types that do not behave in the same way as the manufacturers’ setup beads. One can perform *Rmax* scanning through the DCD and record the findings for each cell type. These can then be used as a starting reference in future sorts for those cell types.

*Rmax* is useful in investigating instrument issues, in particular fluidic instability affecting droplet generation and sort electronics failure. Basic Protocol 2 should be used to assess this. Fluidic instabilities can be subtle; for example, slow changes in the position of the breakoff point (BOP) can be caused by several factors, including air bubbles or dirt in the nozzle, flow cell, or sheath line; temperature changes of the sheath; ill‐fitting nozzle tips; vibration; and pressure fluctuations.

Downstream processes such as PCR and single‐cell transcriptome sequencing (scRNA‐seq) sometimes require accurate cell counts. *Rmax*, with its ratiometric determination of sort performance, can be used to better calculate the actual number of sorted cells in the collection vessel taking recovery into consideration. Basic Protocol 3, for estimating sorted cell number by *Rmax*, can be performed to calculate a more accurate count, or alternatively to adjust the sort time to reach a defined sort count. Please note that the event rate may fluctuate during a sort; therefore, to get a better estimate of the time the sort will need to reach a given target number, we recommend taking CSC samples at regular intervals during the sort, averaging *Rmax* results to calculate and adjust the sort time.

In some sorters, automated sort‐monitor electronics track the physical position of the jet's BOP and adjust the amplitude of the piezo oscillation to ensure that its position is maintained rather than the DCD time. In situations where changes in pressure occur, which affect jet velocity and where the drop generation is not at its maximal growth rate, BOP adjustments by the sort monitor electronics can lead to a loss of recovery. BOP changes will cause a mismatch between the predefined DCD and the particle arrival time at the BOP (Petersen & van den Engh, 2003). The *Rmax* value will decrease in such circumstances, and the operator can judge whether to use the automated sort‐monitoring electronics or not.

## Basic Protocol 1 EVALUATING SORTER SETUP WITH *Rmax*

Assessing whether the instrument is working correctly is vital for a successful sort. It gives the operator confidence that the sorter is working as specified and allows the early detection and troubleshooting of problems derived from both sample preparation and the hardware, including the electronic, optical, and fluidic issues that may arise during the operation. The *Rmax* protocol described here directly evaluates sort performance in terms of recovery using flow cytometry beads—assumed to be a near‐perfect reagent (i.e., with homogeneous suspensions and stochastic interparticle intervals). To avoid any biases in the method covered in this protocol, the target and non‐target particles used for *Rmax* calculation must be of the same type and size, differing only in their excitation/emission fluorescence, so that they behave similarly when running through the sorter. To ensure the stability of the sort during *Rmax* evaluation, it is important to set the droplet generation electronics of the sorter to get the shortest BOP that is insensitive to small changes in the droplet frequency (Petersen & van den Engh, 2003).

**Figure 1:**
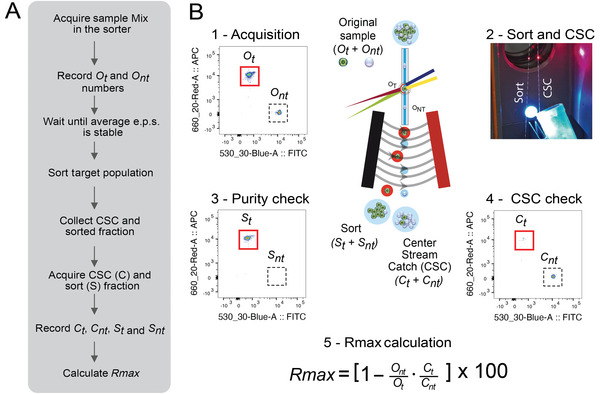
Summary of *Rmax* protocol. (**A**) *Rmax* method workflow and main steps. e.p.s., events per second. (**B**) Example of *Rmax* calculation while sorting APC BD CaliBRITE^TM^ beads (*t*, target) from an original sample (*O*) containing a mix of FITC (*nt*, non‐target) and APC BD CaliBRITE^TM^ beads. (1) After the calculation of the DCD according to the manufacturer's setup instructions, an acquisition file of the *O* sample was recorded to be used for the sorter evaluation. (2) Sort deflections and CSC fractions were collected during the sort; CSC collection into a precoated fresh polypropylene tube was done while sorting the target population. (3) Optionally, the sort fraction can be reacquired to assess the purity of the sort. (4) CSC was also acquired and the number of target and non‐target particles in the original and CSC fractions were entered into the *Rmax* equation (5) to calculate the maximum recovery for those sorting conditions.

The *Rmax* method workflow, main steps, and example of calculation are illustrated in Figure 1. A mix of two fluorescent beads of the same size and material is acquired in the cell sorter after setup and calculation of the DCD according to the manufacturer's instructions. Comprehensive gates around single fluorescent beads are drawn and one of the populations is set as the sort target. In the example in Figure 1B, we use BD CaliBRITE^TM^ APC beads as the target population and BD CaliBRITE^TM^ FITC beads as the non‐target population. The number of gated target (*t*) and non‐target (*nt)* beads in this pre‐sort or original (*O*) sample is recorded. Sort deflections and CSC fractions are collected while sorting the target beads (Fig. 1B1). The CSC is acquired (Fig. 1B4), and the numbers of target and non‐target particles are recorded. The target and non‐target events in the original and CSC samples are used to calculate *Rmax* based on Equation 1 (Fig. 1B5). *Rmax* represents the maximum sort recovery for a given set of sorting conditions.

The method determines the maximum recovery that can be achieved for a given instrument configuration and setup (pressure, nozzle size, optical layout, DCD, etc.). In well‐performing instruments, this procedure only needs to be run once for each configuration and at daily setup as a check on instrument performance. Based on our experience, when using an ideal sample such as the bead mixture stated on this protocol, *Rmax* should be >90% for the instrument to be considered to be working properly.

### Materials

1× phosphate‐buffered saline (PBS; ThermoFisher, cat. no. A1286301)
PBS/2% BSA solution (see recipe)
BD CaliBRITE^TM^ beads: BD CaliBRITE^TM^ 2 colors (cat. no. 349502); BD CaliBRITE^TM^ 3‐colors (cat. no. 340486); and BD CaliBRITE^TM^ APC Beads (cat. no. 340487), using two different fluorescent beads for target and non‐target populations (we use 6‐µm FITC and APC beads here, but in general, any two set of beads with different fluorescence but of the same size and material are suitable)
Detergent‐based cleaning solution such as CoulterClenz (Beckman Coulter, cat. no. 8448222) or 10% CONTRAD (Decon Labs, cat. no. 1002)

Cell sorter
50‐ml polypropylene tube
Transfer pipets
Laboratory vortex or sonication bath
7 × 5‐ml round‐bottom tubes polypropylene (Becton Dickinson GmbH, cat. no. 352063)
Centrifuge
5‐ml tube centrifuge adaptors

#### Equipment setup

**Figure 2:**
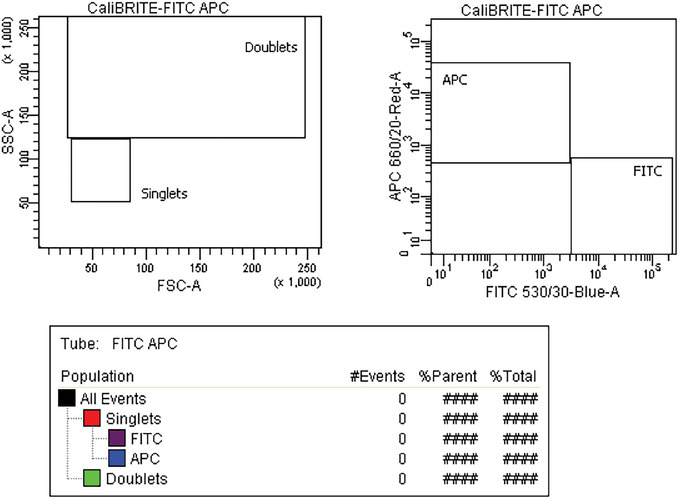
Example of representative plots and gating strategy to be used for *Rmax* protocol.

1 Confirm that cell sorter optical alignment is within manufacturer specifications.
2 Follow manufacturer's recommended procedure to set up the sorter and perform DCD determination as per manufacturer instructions.
3 Create an experimental layout as shown in Figure 2.

#### Bead preparation

We recommend using a mix of two different fluorescent beads rather than a mixture of fluorescent and blank beads to evaluate Rmax. The former allows better discrimination of some doublets, as they will appear as double‐positive particles in a bivariate plot of their fluorescence.

4 To prepare the sort tube, add 0.5 ml PBS and 0.5 ml PBS/2% BSA solution to a 5‐ml polypropylene tube to make a final concentration of 1% BSA in PBS (PBS/1% BSA).
  For this protocol, a total of 10 ml PBS and 50 ml of PBS/2% BSA solution will be needed, along with seven 5‐ml round‐bottom polypropylene tubes, including a tube for the original sort sample, and replicate tubes for the collection of sort deflections and CSC (three of each).
5 Ensure the stocks of FITC and APC CaliBRITE^TM^ beads are well mixed by vortexing or sonicating them.
6 Add 2 drops of each CaliBRITE^TM^ bead to the sort tube (from step 4), to give an approximately 1:1 mix of beads to PBS/1% BSA.
7 Vortex or use gentle sonication to minimize clumps.
8 To prepare for the CSC collection, precoat three 5‐ml polypropylene tubes with 4 ml PBS/2% BSA for 30 min at 4°C and discard the volume after the incubation. Add another 1 ml PBS/2% BSA solution to each precoated tube. Label the tubes as CSC1, CSC2, and CSC3.

#### Bead mix acquisition, gating strategy, and sort setup

**Figure 3:**
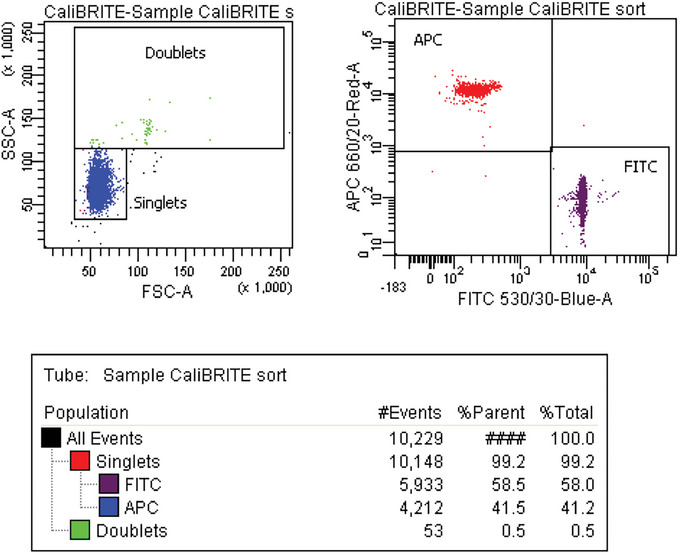
Acquisition of mixed bead sample (original sample).

9 Run the bead mix in the sorter and set the acquisition limit to save 10,000 total events.
10 Set the FSC as the acquisition triggering parameter.
11 Adjust the FSC detector gain and threshold values to limit the collection of debris and allow the acquisition of all the CaliBRITE^TM^ beads.
  Debris will cause soft or hard aborts in Purity precision mode, affecting the calculation of Rmax.
12 Draw and adjust gates in FSC Height versus Area bivariate plots to capture singlet and doublet events, as shown in Figure 3, and adjust the boundaries of the singlet (“beads”) gate to include all the events within the CaliBRITE^TM^ bead singlet population. The percentage of doublets should ideally be <1%.
  If the percentage of doublets is >1%, sonicate the bead mix for 5 min and re‐acquire. If the percentage of doublets is still too high, sort total singlets into a different tube, and use this new tube as the original sample.

**Figure 4:**
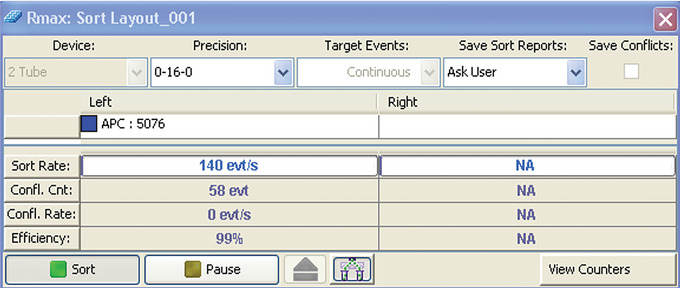
Sort layout example (BD FACSAria IIu cell sorter).

13 Set fluorescence axis to log or biexponential display and adjust detector gain/voltage settings to bring all events off axis.
14 Create and adjust gates around each fluorescent population in an APC versus FITC bivariate plot to ensure that all the events within each CaliBRITE^TM^ bead population are included (Fig. 3).
15 Select one of the populations to sort as “target,” gating on all of the target population, and assign it to one of the outer sort streams.
  The CaliBRITE^TM^ population excluded from the sort gate is henceforth defined as the “non‐target” population.
16 Define target sort limit to “unlimited” or “continuous” sort. Alternatively, set a very large target sort limit (e.g., 10,000,000).
17 Set the sort mode to Purity 1‐drop mode or equivalent. Check the instrument manual to ensure that you are using the correct sort mode. See example of a sort window in Figure 4.
  We recommend the frequently used Purity 1‐drop mode for evaluations of instrument performance with Rmax because it ensures the exclusion of non‐target particles, leading to very high purity and the deflection of one drop per sort decision regardless of target particle position within the drop, thereby enabling us to better assess the accuracy of the DCD.

18 Label triplicate 5‐ml polypropylene tubes (S1, S2, and S3) for the collection of the target sort deflections.
19 Add 0.5 ml PBS and 0.5 ml PBS/2% BSA solution to each sort collection replica tube.
20 Place the sort collection tube in the collection device unit.
21 Start the sort.
22 While sorting, adjust the flow rate to maximize the number of events per second (eps) while assuring the sort efficiency remains ≥99%. Accumulate enough events to fully visualize the boundaries of each population distribution, and adjust gates as necessary to include all singlet and fluorescence events.
  If, at minimum flow rates, the sort efficiency does not increase to ≥99%, that may be due to the bead mix being too concentrated. In this case, take note of the total events per second, stop the sort, remove the sample tube, and dilute the bead mix proportionally with PBS/1% BSA to achieve an event rate close to 1/10th‐1/20th of the droplet generation frequency.

#### Collecting the center stream

CAUTION: A risk assessment must be performed before proceeding. You must follow all local safety rules, including local laser safety rules, when collecting the CSC. Some instruments may require the laser interlocks or the sort chamber interlock to be bypassed in order to collect CSC fractions. There is a potential for electrocution from the deflection plates, and proper steps outlined by your risk assessment and local safety rules must be followed. If unsure, do not proceed and contact the instrument manufacturer for advice. You do this at your own risk; no liability is taken nor implied by the authors or the journal.

23 While the sort is in progress, collect the CSC by placing one of the CSC collection tubes (from step 8) below the deflection plates and under the center (sort waste) stream while avoiding capturing the sorted fraction deflections. Alternatively, adjust the center stream charge to deflect the center stream in the opposite direction from the deflected sort deflections and into the CSC collection tube (see Supplemental Table 1 for instrument‐specific CSC collection).
24 Continue to collect the center stream until the CSC tube is almost full, carefully avoiding cross‐contamination with the sort fraction.
  To reduce contamination with the sort stream during the collection of the CSC, increase the sort drop deflection angle, keeping it clear of the CSC collection tube before initiating collection.
25 Remove the CSC and sort tubes.
  With some instruments, such as the Beckman Coulter CytoFLEX SRT, this may require pausing the sort temporarily. If this is the case, resume the sort after removing the CSC and sort tubes and wait until the number of events per second stabilizes before continuing with the next steps.
26 Repeat steps 23‐25 to collect two additional replica tubes for the CSC and sort fraction.
27 Stop the sort.

#### Analysis of sorted and CSC fractions

**Figure 5:**
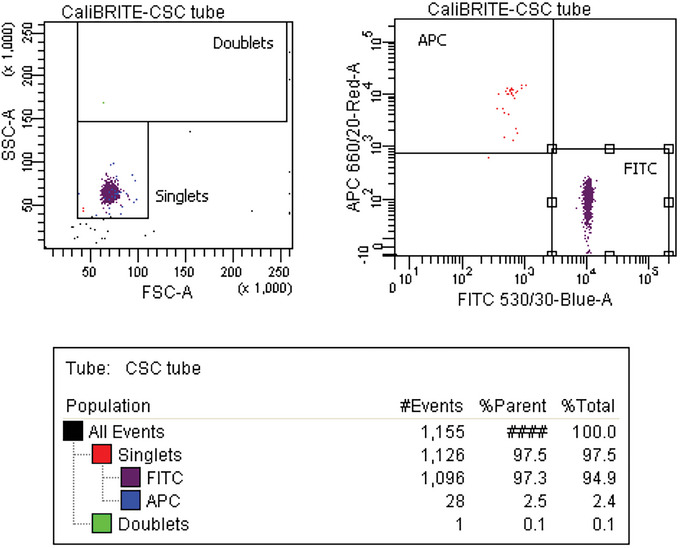
Acquisition of the CSC tube.

28 Centrifuge all CSC tubes for 3 min at 300 × *g*, room temperature.
29 After centrifugation, discard most of the supernatant by carefully pipetting, leaving ∼300 μl at the bottom of each tube.
30 Remove the original bead mixture from the sorter sample station and backflush the sample line for 1 min or run several automatic backflush cycles to remove beads from the sample line.
31 Acquire a tube with cleaning solution for at least 3 min at a high flow rate until there are no residual beads left in the sample line. After that, backflush the sample line for 1 min or run several automatic backflush cycles.
32 Acquire a tube with fresh Milli‐Q water for 1 min to eliminate remnants of the cleaning solution and to confirm the absence of carried over beads.
33 Sequentially load and record the CSC replica tubes, aiming to acquire at least 1000 events from the target population. Alternatively, if near the *Rmax* maximum, collect as many target events as you can (Fig. 5).
  Rmax calculation is critically affected by Poisson counting errors and the acquisition of low counts for the CSC target events, representing, under normal sort conditions, the rarest subset in the CSC. You can safely record all CSC tubes at maximum flow rate for expedient process (i.e., flow rate 11 on a BD FACSAria). Keep in mind that you may need to adjust the FSC × SSC gate to include all beads because as the core sample stream widens, you may lose consistency in scatter measurements; typically, no noticeable changes are observed on the fluorescence channels (see Rmax calculation and sources of error under Critical Parameters and Troubleshooting).

**Figure 6:**
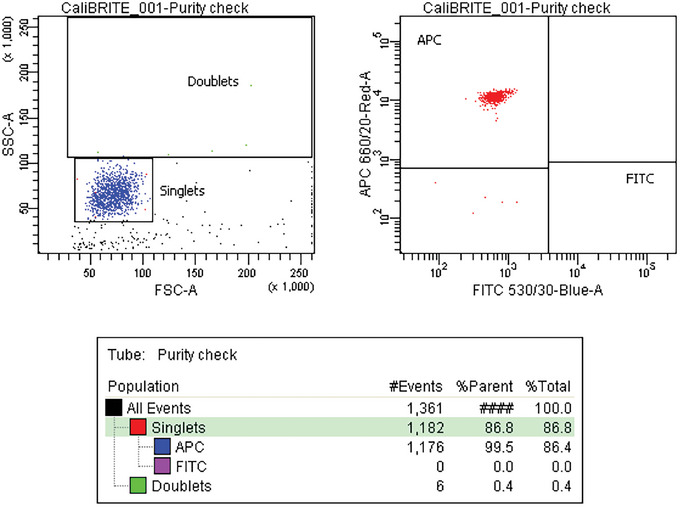
Acquisition of the sorted tube and purity check.

34 *Optional*: To assess the purity of the sort, repeat steps 31‐33 with the sorted fraction and analyze the sorted tubes, saving at least 1000 events of the target particles (Fig. 6).

#### Calculating Rmax

35 Gather the number of events for the target and non‐target populations in the original sample and in the acquired CSC. In the examples shown in Figures 3 and 5, these values are: *O_t_* (APC^+^) = 5,933; *O_nt_* (FITC^+^) = 4,212; *C_t_* (APC^+^) = 28; and *C_nt_* (FITC^+^) = 1,096.
36 Perform a manual *Rmax* calculation based on Equation 1 and using the values gathered in step 35.
$$Rmax_{p\to 100\%}=1-\frac{4,212}{5,933}\cdot \frac{28}{1,096}$$
$$Rmax_{p\to 100\%}=0.982$$
  Equation 1 is used when the target purity in the sort fraction is close to 100%. It can be shown that, for a 1:1 ratio of target to non‐target populations in the original sample, this equation can be used if target purity in the sorted fractions is >95%. Under these conditions, Eq. 1 can be used with <1% error in the value of Rmax (Riddell et al., 2015). If the sorted target purity is <95%, further investigation is required (see Critical Parameters and Troubleshooting section).

## Basic Protocol 2 FINDING THE MAXIMUM *Rmax*: SCANNING OVER THE DROP CHARGE DELAY

This is a useful method to characterize the sorter either when the instrument is first installed or if there are indications that the instrument is not behaving to specification. This method scans sort recovery, with *Rmax* calculated in sorts run at the drop charge delay (DCD) defined following manufacturer's recommended procedure and at additional manually entered DCDs covering the extent of a drop below and above the calculated DCD. This recovery scanning checks whether *Rmax* values lower than the sort efficiency reported for a given set of sort conditions and manufacturer‐recommended DCD setup are due to a poorly determined DCD or to an instrument malfunction. This protocol enables the identification of optimum DCD values as those providing the maximum value for *Rmax*. The protocol requires the same materials as Basic Protocol 1, with quantities and volumes of some items changed as described in the annotation to step 1.

*NOTE*: We do not sample the purity in this protocol.

**Figure 7:**
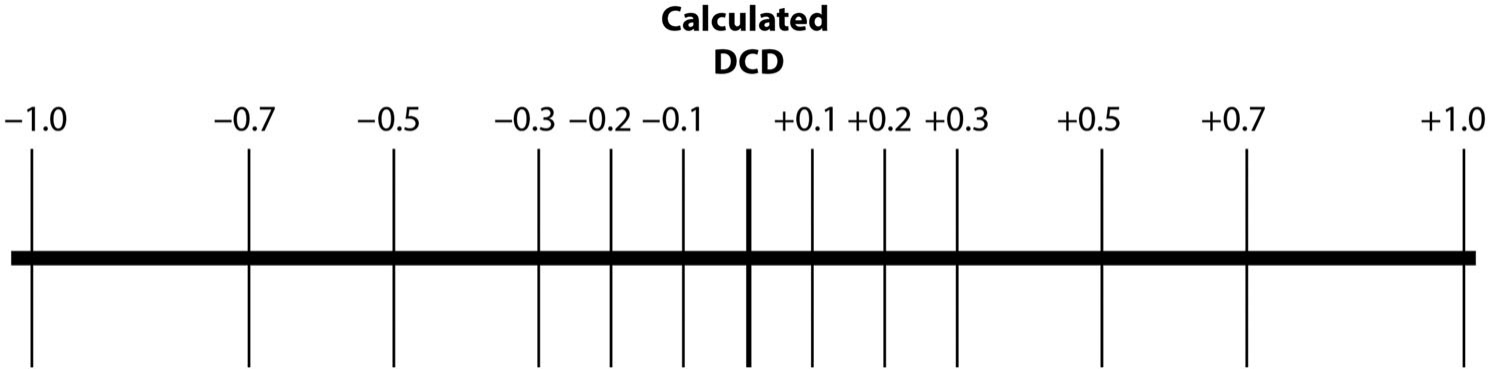
Example of DCD scanning values over the manufacturer‐defined DCD.

1 Follow steps 1‐17 of Basic Protocol 1.
  For this protocol, a total of 20 ml PBS and 500 ml BSA/2% solution will be needed, along with 84 5‐ml round‐bottom polypropylene tubes.
2 Record the DCD reported by the instrument at the end of the setup and calculate DCD values spanning from 1 drop below to 1 drop above this predefined delay. We suggest using 13 DCD values including the instrument‐determined DCD, as shown in Figure 7.
  In most cell sorters, the DCD, representing the time from sort target classification at the interrogation point to sort deflection at the BOP, is expressed in droplet (period) equivalents, making it easy to calculate the range of delays for this protocol. In cell sorters such as the Cytek^®^ Aurora CS, DCD values are reported directly in units of time. To define DCD scanning values for this protocol in cases like this, first calculate the droplet period as the inverse of the droplet generation frequency (expressed in Hz or s^–1^). Convert the calculated value from seconds into the time units used by the instrument to report the DCD. This calculated time can be subtracted from or added to the reported DCD to obtain the lowest and highest DCD scanning values, respectively. Fractions of the drop period can be added to or subtracted from the reported DCD to derive the entire range of delay values shown in Figure 7.

3 Label triplicate tubes for the collection of the sort (purity) and CSC fractions for each of the DCD time points (see the comment in Fig. 7).
4 You should now have 78 tubes in total, 39 tubes for the CSC and 39 tubes for the sort collection. Place them in DCD order in a suitable rack.
5 Set the DCD to one drop below the instrument calculated DCD. This is the starting point of the scan.
6 Follow Basic Protocol 1, steps 19‐27.
7 Increase the DCD value one step and repeat steps 19‐27 of Basic Protocol 1. Repeat twelve more times to cover all the DCD scanning values (13 in total).
  You'll be measuring Rmax for sorts run at different DCD by stepwise increments and acquire triplicates at each step until all the tubes for the curve data points are collected. Use shorter DCD intervals around the DCD determined according to manufacturer instructions.
8 Perform steps 28‐34 of Basic Protocol 1 to process and analyze all the CSC and sort collection tubes.

#### Calculating Rmax

**Figure 8:**
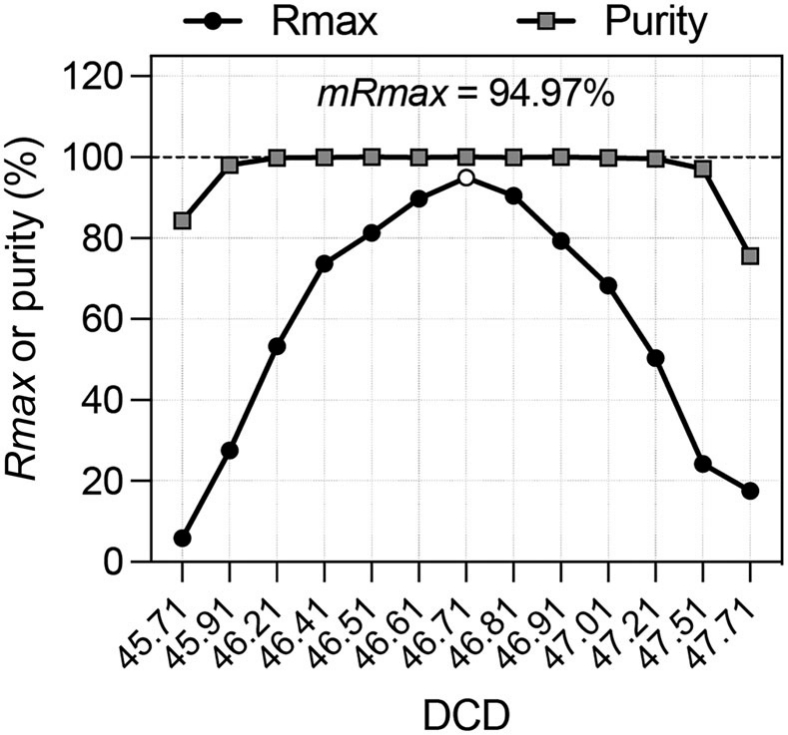
Example of *Rmax* versus purity scanning over the DCD. The white circle indicates the *Rmax* maximum value (*mRmax*), corresponding to the optimum DCD the sorter should be set to.

9 Perform steps 35‐36 of Basic Protocol 1 to calculate *Rmax* and sort purity values for each DCD point.
10 Plot purity and *Rmax* versus DCD values in Excel or other data analysis software and generate best‐fitting curves (Fig. 8). The optimum DCD value is the one giving the maximum value of *Rmax*.

## FINDING THE MAXIMUM *Rmax* FOR CELLS: SCANNING OVER THE DROP CHARGE DELAY

In a sorter, properly prepared beads generally flow with a probability of coincidences following a Poisson distribution. They can be considered “ideal particles” to evaluate the instrument. In contrast, flowing cell coincidences do not necessarily follow a Poisson distribution (Lindmo & Fundingsrud, 1981), which can lead to *Rmax* values <80% in sort modes aimed at high purity. It is nonetheless useful to evaluate *Rmax* on cells across different drop charge delays (DCDs) to identify the optimum value providing maximum recovery, thus ensuring best conditions for the sort. This protocol scans the ±3/10th of a drop around the manufacturer's determined DCD. A total of seven DCDs are evaluated in triplicate sorts to identify the optimum DCD maximizing the recovery of the target cells. The protocol requires generally the same materials as Basic Protocol 1, with quantities and volumes of some items changed as described in the annotation to step 3.

*NOTE*: We do not sample the purity in this protocol.

#### Additional Materials (also see Basic Protocol 1)

Cell suspension for cell sorting

1 Setup the sorter and calculate the DCD following manufacturer‐recommended procedures (Basic Protocol 1, steps 1 and 2).
2 Record the DCD reported by the instrument.
  For the Rmax scanning, we suggest using seven DCD values: three above and three below the DCD defined by the manufacturer's recommended procedure, ±3/10th of a drop, as shown in Figure 7.
3 Label triplicate tubes for the collection of the CSC fractions of each of DCD time point. You should now have 21 tubes in total. Place them in DCD order in a suitable rack and add 0.5 ml PBS/2% BSA solution to each 5 ml polypropylene tube.
  For this protocol, a total of 20 ml PBS and 500 ml PBS/2% BSA solution will be needed, along with 21 5‐ml round‐bottom polypropylene tubes.
4 Acquire the cell sample and set up plots, gates, and target sort logic.
5 Start to sort and wait until the total event rate stabilizes.
6 Follow Basic Protocol steps 23 and 24 to collect the CSC, taking care not to contaminate it with the sorted stream, and repeat for each replica.
7 Stop the sort.
8 Manually change the DCD value and follow Basic Protocol 1, steps 20‐27, six more times to collect the CSC tubes for the remaining DCD values.
9 Spin down your cells with a centrifuge using settings (*g* force, time, and temperature) appropriate for your cells, and follow Basic Protocol 1, steps 29‐34, to analyze the CSC and record the target and non‐target counts in each of the CSC collection tubes.
10 Follow Basic Protocol 1, steps 35 and 36, to calculate *Rmax* for each replicate.
  We do not sample the purity in this protocol.
11 Plot the average *Rmax* against DCD values as seen in Figure 8 and identify the optimum DCD as the value providing the highest *Rmax* value.

## Basic Protocol 3 ESTIMATING SORTED CELL NUMBER WITH *Rmax*

During sorting, it is assumed that deflected drops always contain target cells. If this were true, the sort counters on the instrument would give an accurate report of the number of cells sorted, but this has been shown to not always be the case (Riddell et al., 2015). In experiments in which sorted cell number is just as important as sort purity, *Rmax* can be used to get a better accuracy of the true sort count. In long sorts, collecting at least three repeated CSC samples to calculate an *Rmax* average will improve the precision still further. When the sort has finished, multiplying an average *Rmax* by the final instrument sort count will give a better approximation to the true sort count. The operator can then decide whether to sort more targets to get the desired total value. The protocol requires the same materials as Basic Protocol 1, with quantities and volumes of some items changed as described in the annotation to step 3.

### Additional Materials (also see Basic Protocol 1)

Cell suspension for cell sorting

1 Set up the sorter according to the manufacturer's protocol and confirm that the instrument optical alignment is within manufacturer specifications.
2 Follow manufacturer's recommended procedure to set up the sorter and perform DCD determination as per manufacturer instructions.
3 Begin sorting the cells of interest and adjust the flow rate.
  For this protocol, a total of 20 ml PBS and 50 ml PBS/2% BSA solution will be needed, along with three 5‐ml round‐bottom polypropylene tubes.
4 Prepare the CSC tubes as described Basic Protocol 1, step 19.
5 Collect the CSC, taking care not to contaminate it with the sorted stream.
6 After CSC collection, stop the sort temporarily and record the sort count on the instrument counter (*I_t_*), the reported sort efficiency (*E*), the original target (*O_t_*), and the original non‐target (*O_nt_*).
7 Follow Basic Protocol 1, steps 30‐33, to clean the sample line and acquire and analyze the CSC tubes.
  This can also be done on a separate analyzer of suitable type.
8 Record the *C_t_* and *C_nt_* values.
9 Replace the sorting sample and continue with the sort.
10 Calculate *Rmax* using Eq. 1 (from Basic Protocol 1, step 35) and record it.
11 If you wish to calculate *Rmax* for CSC replica tubes, repeat steps 4‐10 for replicates and calculate the average *Rmax* ($\bar{Rmax}$ *)*.
12a If the reported sort efficiency was near 100%, use Eq. 2 to estimate recovery (*N_Est_*):
(2) $$N_{Est}=I_{t}\cdot \bar{\dot{Rmax}}$$
12b In cases where the reported sort efficiency *E* was <99%, Eq. 2 becomes:
(3) $$N_{Est}=I_{t}\cdot \bar{Rmax}/E\;\left(E\;>\;0\right)$$
13 Optionally, if accuracy in sort number is critical for downstream processing, you can calculate the extra amount of sort time needed to achieve that number:
(4) $$T_{st}=\frac{N_{t}-N_{Est}}{S}$$

where $T_{st}$ is the “total sort time” needed to reach the desired sort target number in minutes, $N_{t}$ is the target number required, and *S* is the average sorts per second.

## REAGENTS AND SOLUTIONS

### PBS/2% BSA

Dilute 1 g bovine serum albumin (BSA, Merck, cat. no. 810533) in 50 ml PBS and then filter through a sterile single‐use 0.2‐µm syringe filter (Sartorius, Minisart^®^, cat. no. 16534‐K).

The solution can be freshly prepared on the same day, ahead of the Rmax protocols, or days in advance. It can be stored at 4°C for up to 1 week, or stored as aliquots kept at −20°C for future use.

## COMMENTARY

### Background Information

To isolate pure cell populations, cell sorters have sophisticated electronics able to identify, classify, and handle coincident events, including those between target particles (satisfying the defined sort logic) and contaminants. A coincidence occurs when particles arrive very close to each other at the laser interrogation point, either within the duty cycle of the electronics (known as hardware or *hard* coincidence) or close enough to later fall within the boundaries of the deflected volume (known as *drop* or *soft* coincidence). In a perfectly operating instrument, all the hard coincidences and some of the soft coincidences, depending on sort mode, will lead to sort aborts and loss of the target particle as it is discarded towards the waste with the center stream. The frequency of sort aborts increases with the rate of acquisition‐triggering events, impacting hard and soft coincidences. The likelihood of soft coincidences increases with the average droplet occupancy in the jet, calculated as the ratio between the rate of acquisition‐triggering events and the piezo oscillating frequency. Additionally, the probability of aborts due to soft coincidences compromising purity, and therefore including target and non‐target particles, increases with the reduction in the frequency of target particles out of the total triggering events.

The impact of soft coincidences on sort decision varies according to the selected sort mode, being a combination of sort precision level and drop envelope. Single‐mode sorts avoid all soft coincidences, guaranteeing the deflection of droplets containing exclusively one target cell, which results in sorted fractions with high purity and accurate counts but reduced recoveries. In Enrich‐mode sorts, all droplets containing target particles are deflected regardless of coincident contaminants, increasing overall sort yield but sacrificing purity. Purify or Purity is the sort mode most frequently used in cell sorting, enabling the deflection of droplets containing one or more target particles in the absence of coincident contaminants, thus ensuring high purity and recovery. The drop envelope accounts for the number of deflected drops, the location or phase of the target within the drop, and the uncertainty in the target's final allocation when close to the drop boundaries.

Although *Rmax*, via its general equation (Riddell et al., 2015), can be used to calculate the recovery of sorts carried under any sort mode, we recommend the frequently used Purity 1‐drop mode for evaluations of instrument performance because it ensures the exclusion of non‐target particles, leading to very high purity and thus enabling the use of *Rmax* Equation 1 without mandatory re‐sampling of the sort fraction.

In the absence of reports on sort efficiency, an indication of instrument fitness and sample quality is how closely *Rmax* values match the theoretical maximum expected recovery. In droplet sorters, the distribution of non‐interactive monodisperse particles along drop equivalent segments of the jet will ideally follow a stochastic Poisson probability profile with a proportion of drops containing 0, 1, 2, …, *n* particles. Additionally, the probability of a target particle being sorted depends on the relative frequencies of target and non‐target events for each of the coincidence scenarios (*n* > 2), and these can be modeled using the binomial distribution. The outcome of a sort in terms of recovery (Single‐cell and Purify modes) and purity (Enrich mode) can be modeled and anticipated this way as a function of the average event rate, the droplet generation frequency, and the frequency of the target population out of total triggering events in the original sample.

The theoretical maximum recovery defined, and the sort efficiency reported by the instrument during a sort, are based on the analysis of events captured at the interrogation point. Both assume flawless downstream processes and do not account for additional sources of particle loss. A critical factor affecting recovery is the DCD defined empirically with beads during sorter setup and based on the beads’ traveling time from interrogation point to BOP, assumed to match that of the target cells. Additional assumptions are being made in the theoretical maximum recovery model: (1) that a Poisson distribution of monodisperse particles along the jet is the sole contributor to the expected frequency of coincidences (Watson, 1991, p. 12); (2) that the speed of particles inside the jet or cuvette and their traveling time to the deflection point are uniform, with particle pulses maintaining their relative position, or phase, against the surrounding jet's disturbance at all points along the jet; and (3) that there is no interference of particles with the jet's disturbance and the BOP position, affecting the charge allocation per drop and sort stream deflection angles.

**Figure 9:**
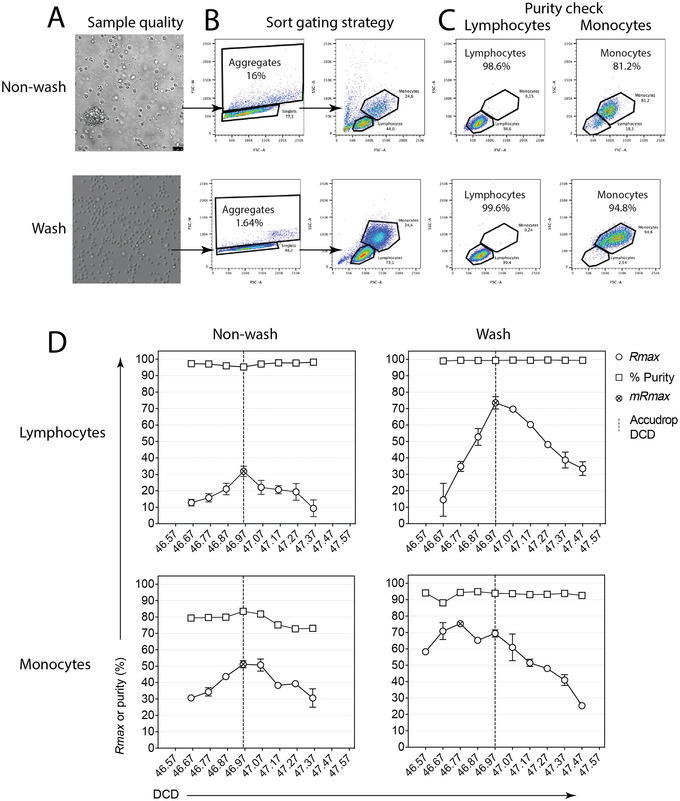
Sorting of BioLegend Veri‐Cells™ lymphocyte and monocyte fractions during evaluations of *Rmax* via Alternate Protocol. (**A**) The original Veri‐Cells sample (“non‐wash,” above), prepared by resuspending cells in BioLegend buffer, was inspected with a Leica confocal microscope, revealing abundant cell clumps and debris. Sample quality substantially improved after two rounds of washing, including the centrifugation of cells resuspended in 5 ml of PBS/2% BSA and 3 mM EDTA, and their final reconstitution in 1 ml of the same buffer (“wash,” below). (**B**) The Veri‐Cells™ original (non‐wash) and improved (wash) samples were analyzed in a BD FACSAria IIu, confirming the reduction in the abundance of debris and aggregates after washing. For the *Rmax* experiments (Alternate Protocol), monocytes and lymphocytes were alternatively sorted based on gates around singlets and scatter profiles using Purity 1‐drop mode. These gates are very close together and contamination can occur near the boundary. (**C**) The analysis of the sorted fractions revealed consistent high purities for sorted lymphocytes and differences in monocyte purity, depending on the original sample. Notice the monocyte (target) contamination with lymphocytes (non‐target) when sorting the non‐washed sample (above, 18.3% lymphocytes) compared to sorts using the sample improved by washing (below, 2.54% lymphocytes). (**D**) Scanning for purity and *Rmax* (mean and error for 3 replicas) when sorting BioLegend Veri‐Cells™ monocytes or lymphocytes at different DCD values, based on Alternative Protocol 2. Sorts with lymphocytes or monocytes were done at several DCD values (from 46.57 to 47.47), including the Accudrop‐defined DCD settings of 46.97, for Veri‐Cells™ washed sort, or 46.96, for Veri‐Cells™ non‐washed sort. Although maximum recoveries (*mRmax*) in lymphocytes are achieved at the manufacturer's defined DCD (Accudrop) in both washed and non‐washed samples, the impact of the nature of the target population and the quality of the samples on sort performance can be appreciated in the observed differences in *mRmax* (*mRmax*, “wash” > “non‐wash”), sort purity (“wash” > “non‐wash”), and optimum DCD (“non‐wash” > “wash”).

The practical limitations of both theoretical recovery predictions and interrogation‐point‐informed reports on efficiency derive partially from the deviation of sorted cells from ideal flow behavior and their interference with the feeble forces driving the droplet generation process. Interparticle intervals for cell suspensions have been shown to not necessarily follow a Poisson distribution (Lindmo & Fundingsrud, 1981), so you cannot always accurately predict recovery using theoretical models based on Poisson‐binomial distributions (Watson, 1991, p. 12). Secondly, for jet‐in‐air droplet sorters, the piezo vibration energy involved in droplet generation is small and aims to synchronize the natural breakup of a jet into droplets led primarily by surface tension. Large enough particles exiting the nozzle, as they relax from hydrodynamic forces and parabolic flow, have the chance to interfere locally with the mild waves concurrently traveling alongside the jet. This interference ultimately influences the BOP position and the consistency in the charge allocated per drop due to a mismatch between break‐off time and the predefined DCD (Stovel, 1977). Finally, the assumption of consistent linear velocity among particles as they flow from interrogation to deflection points contrasts with changes in particle velocity observed in jet‐in‐air cell sorters following the parabolic flow relaxation that occurs after nozzle exit. Differences in the speed of particles within heterogeneous samples influence their respective BOP arrival times (Van Dilla et al., 1985, pp.111), explaining differences in optimum DCD, such as those observed in DCD scanning experiments with *Rmax* using sort target particles of different diameters (see Critical Parameters and Troubleshooting and Fig. 9D).

The above examples illustrate sample‐related (instrument‐extrinsic) factors affecting sort recovery, which can be minimized to an extent via the optimization of sample preparation and suspension buffer. In some scenarios, their impact on sort recoveries will be unavoidable, a consequence of target cell sizes relative to the jet's diameter or the cell's inherent propensity to form aggregates. These, however, do not reflect on the instrument performance or indicate its malfunction. It is critical for a sort operator to be able to distinguish extrinsic from intrinsic sources leading to experienced poor recoveries. *Rmax* can be used to troubleshoot recovery issues due to sample preparation and to differentiate these from instrument‐related factors. For instance, this can be done by comparing *Rmax* results from the instrument setup with beads as a model of an ideal sample (Basic Protocol 1), against *Rmax* results with cells (Basic Protocol 3). Malfunction of the sort electronics will give a consistently low *Rmax* for both beads and cells, irrespective of sort efficiency reports or sample preparation (Riddell et al., 2015). This may be due to defective sort classification or droplet charge timing with either the software or electronics boards compromised and will require a field service engineer visit. However, a reduction in calculated *Rmax* only in cell sorts can result from excessive cell death, cell clumping and aggregate exclusion, differences between bead and cell size, hydrodynamic properties influencing drop formation, BOP stability, optimum DCD, or cell loss due to improper handling of the cell fractions or cell adherence to tube walls.

Overlooked by both theoretical recovery calculations and reported sort efficiencies, a major issue that can cause target particles to be lost to waste during sorts is inaccuracy of the DCD settings. In jet sorters, most methods used to set the DCD include the counting of a sorted population. Historically, the calculation of DCD in drops equivalent involved measuring the distance from the first laser interrogation point to the center of the last attached drop; this value was then divided by the wavelength of the piezo oscillation. A slightly more complicated calculation is needed for sense‐in‐cuvette droplet sorters (Van Dilla et al., 1985). This gives a rough estimate of the DCD, with a precise definition requiring the empirical evaluation of target particle recovery during sorting over a given range of discrete DCD values. Instrument‐embedded DCD calculation workflows can include semi‐automated Puddles‐Slide‐based methods (Beckman Coulter, 2017; Lazebnik et al., 1992). In the Beckman Coulter CytoFLEX SRT, the automatic calculation of DCD is achieved via a hybrid approach that includes first measuring QC bead travel time (DD1) from the triggering laser at the cuvette to a dedicated laser (T1) interrogating particles at the jet a few drops before the BOP. DD1 is added to the remaining transit time from T1 laser interrogation to BOP, calculated based on strobe image analysis (Beckman Coulter, 2022). A fully automated approach for DCD calculation is also used in the Becton Dickinson Accudrop^™^ and other systems (Bio‐Rad, 2013; Norton, 2002).

Manually confirming the accuracy of the critical DCD parameter typically requires repeated sampling and the absolute counting of sorted target particles to identify the optimum DCD value at which a best match between the number of sorted target particles reported by the electronics and the number of deflected particles in the collection device is achieved. This leads to potentially high errors associated with absolute counting, concentration, and volume estimations (Dacie & Lewis, 1984; Kirkman‐Brown & Björndahl, 2009). Counting methods based on the Coulter principle or cytometric counting beads (Lapping et al., 1972; Nicholson et al., 1997; Pegg & Antcliff, 1965) offer better accuracy due to the larger number of particles being sampled; however, they may still suffer from volume measurement errors. The ratiometric nature of *Rmax* and its focus on target loss estimation via CSC sampling and analysis circumvent the above absolute counting issues, providing a direct measure of the maximum recovery expected for a given instrument configuration (nozzle size, sheath pressure, and drop‐drive frequency) and sort setup (calculated drop delay, sort‐precision mode, and sort efficiency). The Basic Protocol 2 described here can be used to assess the accuracy of the instrument's predefined DCD calculation methods and to define the optimum deflection time for a given instrument configuration, sample, and target particle. The maximum *Rmax* (*mRmax*) can then be used as a reference *Rmax* for that configuration and experimental setup, and a discrepancy between *mRmax* and the daily *Rmax* can be used to quickly identify problems arising with the instrument or the sample. Based on our experience, and due to the strict dependency of recovery on DCD values, changes in *Rmax* can frequently derive from improper deflection timing determination, no matter how slightly.

### Critical Parameters and Troubleshooting

Multiple factors contribute to the success of the *Rmax* protocol and require special attention.

#### Instrument setup

Before running the *Rmax* protocols, it is crucial to guarantee that the sorter is at its best possible operational status, especially when aiming to define performance baselines for a given configuration and set of conditions. Follow the manufacturer's recommendations for instrument and fluidics initialization and sorter setup. Before running the manufacturer‐recommended QC and proceeding to the optimization of sort parameters, test the stability of the observed drop generation pattern at the jet, the position of the BOP, and its geometry (relative position and appearance of main drops and surrounding satellite drops). The consistency and robustness of the above can be confirmed after running several debubbling cycles for the flow cell and the sheath filter. An additional simple test for the absence of air in the fluidics of sort‐in‐air sorters is to transiently switch off the sheath line(s) while looking at the timing of the resulting jet's disappearance. If the jet does not snap off quickly, then it is likely that there are air bubbles in the nozzle assembly, sheath lines, sheath filter, and/or sheath tank. Following recommended cleaning procedures for the sheath lines, flow cell, and nozzle is fundamental. Air bubbles can form from small dirt particles in the nozzle assembly and in the sheath lines. Air bubbles and dirt trapped in the flow cell or nozzle will lead to the disruption of the laminar flow profile, ultimately compromising sort performance. The resulting changes in fluid velocity profile along the jet's cross section can be readily identified via the loss of the axial symmetry of the liquid jet and the drops being generated along it. Clean all the components, especially the nozzle, as specified by the manufacturer's instructions and, if necessary, replace the nozzle, sheath line, filter, and/or tank. When defining performance baselines with *Rmax*, take note of the specific nozzle used for the original baseline definition, as small variations in nozzle manufacturing are typical even among nozzles of the same output diameter specification.

#### Sample preparation

To ensure that cells and beads are not lost to the walls of the plastic sample and collection tubes (Brando et al., 2001), we recommend blocking these with a solution of 2% to 5% BSA dissolved in PBS. Fill the tubes to at least one‐third of their capacity with this solution and roll rotate them on a rotary shaker overnight at 4°C. For other containers, we recommend filling them completely with the BSA solution and incubating overnight at 4°C. Use sterile technique where appropriate.

When performing the *Rmax* protocols, it is important to minimize the frequency of doublets and aggregates in the original pre‐sort sample. If your sample has too many clumps of cells, the coincidence electronics will abort them, and sort target particles will end up in the waste, affecting the recovery. Also, there are limits to the pulse‐processing electronics and their ability to resolve doublets from singlet events. Particles flowing too close together or touching could be misclassified as a singlet event. When sorted, they can separate from each other while undergoing velocity and pressure changes along the nozzle, or downstream in the collection container. For this and other reasons, we recommend the use of bright fluorescent target and non‐target particles such as CaliBRITE™ beads for Basic Protocols 1 and 2. Besides the ability to fully resolve each population based on fluorescence content, an additional benefit is easy identification of potential singlet misclassifications. Doublets between target and non‐target particles disguised as singlets will appear in the fluorescence plot as fake double‐positive events. In contrast, using a fluorescence‐negative particle for the non‐targets will result in some doublets being mistakenly classified as singlet sort targets, compromising the purity of the sort fraction. In the worst‐case scenario, doublets between a fluorescence‐negative target and a fluorescence‐positive non‐target could be classified as non‐target particles and discarded to the waste. The CSC will then be contaminated with the target, compromising the *Rmax* result. Another scenario would be when two fluorescence‐negative non‐target particles are misclassified as a singlet and their autofluorescence sums, causing them to fall into the fluorescence‐positive target gate. This can happen when you are sorting target cells with low levels of expression markers where the population resolves very close to the negatives (non‐target), or when sorting populations based on barely resolving parameters such as scatter (Fig. 9B), where the purity of the sort would be compromised. Instrument sampling error can occur if the diluted CSC sample is acquired at very high differential pressure, causing the core to widen and thereby leading to variations in illumination.

It is therefore important to review the quality of your sample preparation—that is, to do a visual inspection under a microscope to ensure that you have monodisperse cells or particles—before running them on the sorter (Fig. 9A). It is always good to pass samples through a suitable mesh, depending on the nozzle diameter and sample; for example, use a 35‐ or 50‐µm mesh (depending on your sample and nozzle settings) to remove clumps from peripheral blood mononuclear cells. Supplementing the sort sample buffer with 1 to 5 mM EDTA and preincubating the sample for 30 min with 100 µg/ml DNase I, which can also be kept in the solution at a maintenance dose (25‐50 µg/ml) during the sort, can help prevent aggregates.

Small changes to the sample preparation protocol, such as altering incubation times of enzymes or antibodies, can have a dramatic effect on clumping. Proper sample preparation will help minimize aggregates and clumps that affect *Rmax*. We recommend that you check the literature for best practice for your cell type/particle. When assessing the quality of the preparation under a microscope, it is important to consider both total cell number and viability, using trypan blue or another viability dye. Any viability dye will do; the one chosen will depend on the markers—the panel of fluorochromes or fluorescent proteins—used. Also assess the amount of debris present. If there is a lot of debris, increase the number of washes in your protocol. Acquisition‐triggering debris can coincide with monodispersed cells in a flow cytometer, leading to sort aborts, and if the debris has fluorochromes attached, this can affect the classification of the target cells in the sort gate, reducing *Rmax*. Figure 9D shows *Rmax* and purity results obtained when sorting monocytes (BioLegend Veri‐Cells^TM^) in the BD Aria IIu at Accudrop‐defined DCD and surrounding values (DCD scanning, Alternate Protocol 2). Differences in monocyte sort purity, optimum DCD, and maximum *Rmax* were observed for the original Veri‐Cells^TM^ sample (“non‐wash”, with a large frequency of aggregates and debris) compared to the improved “wash” sample (washed twice, resulting in a reduced frequency of aggregates and debris).

#### Sort mode, sample acquisition, and analysis

**Figure 10:**
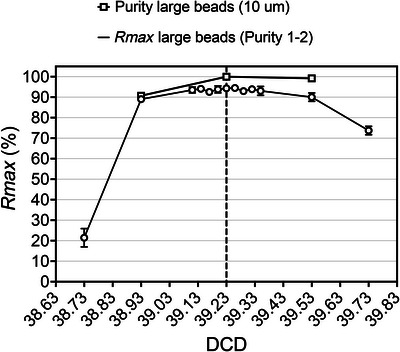
Consistency of *Rmax* around manufacturer's defined DCD optimum in sorts with 1‐2‐drop envelope. Purity and *Rmax* (mean and error for 3 replicas) values in DCD scanning sorts in Bio‐Rad S3 cell sorter following Basic Protocol 1. Instrument setup and the DCD calculation (39.23) were performed based on the manufacturer's recommended procedures. Beckman Coulter Flow‐Check^TM^ Pro Fluorospheres (cat. A69183), consisting of a mixture of fluorescent beads excited by the blue (10 µm), red (6 µm), and violet (3 µm) lasers, were acquired and blue‐laser‐excitable 10‐µm (“large”) beads were chosen as the sort target and sorted using the instrument's default Purity 1‐2 mode. In contrast to DCD scanning experiments using Purity 1‐drop envelope sort modes, this Purity 1‐2 sort produced “flat‐top” *Rmax* curves around the S3 setup DCD, with consistent recovery values from DCD 39.11 to DCD 39.35.

When using *Rmax* to evaluate instrument performance we recommend selecting sort modes with a 1‐drop envelope. This is especially important when using this metric to evaluate the accuracy of DCD values calculated via manufacturer‐recommended procedures or to define optimum DCD leading to maximum recovery for a given sorter configuration and target population. Figure 10 shows the relative independence of *Rmax* values from DCD settings around the manufacturer's defined optimum in Purity sorts with 1‐2‐drop envelope. In all the *Rmax* protocols, it is important to make sure that the sample is properly mixed and the particles are not allowed to settle during the sort. We find that adding 0.5% BSA to the sort buffer improves the *Rmax* in most cases. We hypothesize that this both reduces the “stickiness” of the cells, leading to fewer doublets, and increases the viscosity of the sort sample, helping keep particles in suspension and thus lengthening the particle settling time. If the feature is available in the instrument, enable the automatic stirring or mixing of the sample during the sort, both to reduce the likelihood of aggregates and to guarantee a homogeneous cell suspension leading to consistency in flow rates and original target‐to‐non target ratio. When automatic sample mixing is not possible, it may be necessary to stop the sort and mix the sample manually during *Rmax* evaluations along long sorts.

When acquiring the CSC sample, it is important to set the sample differential pressure to values that do not distort the original position of target and non‐target events relative to the original gates. This impacts *Rmax* and obviously the purity measurements, even when the sort was pure.

#### Rmax calculation and sources of error

**Figure 11:**
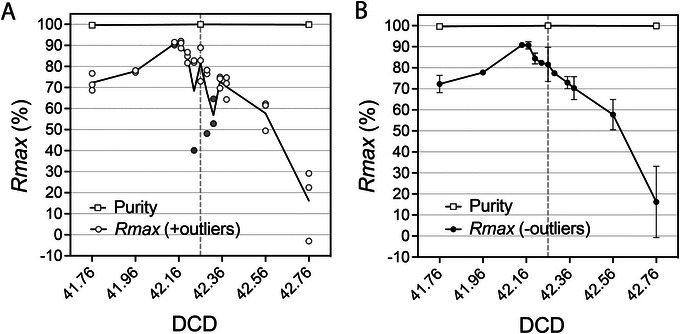
Contamination of the CSC with sort deflections leads to *Rmax* inconsistency and error. (**A**) Purity and *Rmax* (line representing the mean value across 3 replicas, individually shown) values in DCD scanning sorts in BD FACSAria I cell sorter following Basic Protocol 1. Instrument setup and DCD calculation (42.26) were performed based on manufacturer's recommended procedures. Beckman Coulter Flow‐Check^TM^ Pro Fluorospheres (cat. A69183), consisting of a mixture of fluorescent beads excited by blue (10 µm), red (6 µm), and violet (3 µm) lasers, were acquired and the violet‐laser‐excitable 3‐µm beads were sorted as target using 0‐16‐0 Sort mode (Purity 1‐drop). *Rmax* outliers due to contamination of the CSC fraction with the sort deflections are highlighted in grey. (**B**) Same experiment as in **A**; the removal of outliers renders the typical smooth *Rmax* profile of DCD scanning experiments using Purity 1‐drop mode.

Although the ratiometric nature of *Rmax* improves the accuracy of sort recovery assessments by avoiding absolute counting, the procedure can still be affected by error sources related to sample preparation, acquisition, and analysis, as well as CSC collection. Liquid splashes during CSC collection can lead to cross‐contamination of the CSC and sort fractions. An example of *Rmax* outlier due to cross‐contamination during a DCD scan based on Basic Protocol 2 is shown in Figure 11. When collecting the CSC in droplet sorters, place the collection tube and be careful not to contaminate it with the sort stream. Using a flashlight to illuminate the center stream and sort deflections will help. While setting up the sort, increase the angle of the deflected sort stream so that it is sufficiently away from the center stream.

The impact of both total event rate and original target frequencies on sort aborts, and consequently on target recovery, must be taken into consideration when setting up *Rmax* experiments. For sorts with reported efficiencies of <100%, inconsistencies in total event rate along replica sorts used in the *Rmax* protocols will add variability to the resulting *Rmax* values (Riddell et al., 2015). To avoid this, ensure that the flow rate of the particles during the sorts for *Rmax* calculation is low enough to enable very high reported sort efficiencies, while still allowing enough target particles to be acquired for downstream evaluation of CS. Optimum efficiency values near 100% for BD CaliBRITE^TM^ beads and >95% for cells can typically be achieved with total events per second between 1/15th and 1/20th of the droplet‐generation frequency rate for one‐drop sort modes. When running *Rmax* replica sorts at <100% efficiency, and if the sort is paused or halted after the collection of the CSC fraction, ensure that the total events per second plateaus upon resuming the sort and before collecting more of the CSC. The recovery of sorts with reported efficiencies <100% will also be affected by changes in target to non‐target ratios, the definition of the acquisition threshold, and changes in the relative abundance of debris, decreasing the percentage of target particles out of total triggering events and increasing the probability of coincidence‐driven aborts. As aborts are taken into consideration in the calculation of sort efficiencies reported by the instrument, values of *Rmax* closely matching reported efficiency values will be an indication of good overall performance of the instrument. When sorting bead mixes in Recovery mode, and even in Purity mode, as long as sorting efficiency is close to 100%, the optimum *Rmax* values should also be very close to 100%. Larger discrepancies could indicate that the instrument was not properly set up or in perfect condition, or that extrinsic factors such as poor sample quality or physical properties of the target particles are driving them away from a fully Poissonian flow behavior.

Other sources of error in the calculation of *Rmax* include the improper definition of gates, leading to failure to include all of the events within scatter, singlets, and fluorescent target and non‐target populations during the analysis of the original sample ahead of the sort and during the acquisition of the CSC; and differences in fluorescence sensitivity between the cell sorter being evaluated with the *Rmax* protocol and the analyzer chosen for CSC acquisition and analysis. Measurement consistency and instrument sensitivity are critical factors to consider whenever target events are defined via poorly resolving parameters such as scatter, dim particle fluorescence, or fluorescence out of tertiary or secondary cellular markers. Choosing bright, fully resolved fluorescent beads, such as BD CaliBRITE^TM^ beads, for both target and non‐target particles eliminates both issues.

**Figure 12:**
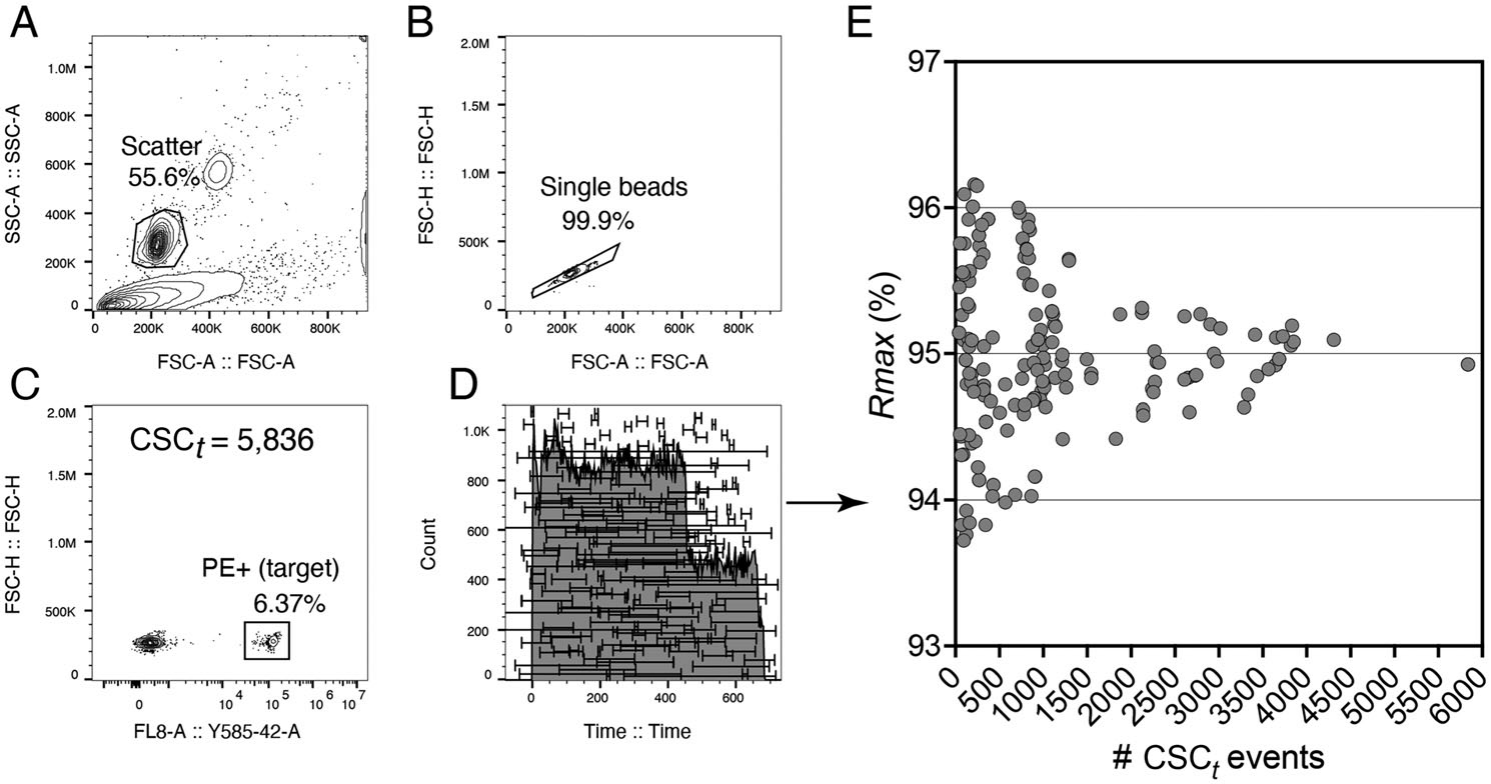
*Rmax* precision is affected by low particle counts. (**A‐C**) Analysis and gating strategy of CSC fractions in *Rmax* experiments (Basic Protocol 1) evaluating the performance of the Beckman Coulter CytoFLEX SRT cell sorter. DCD (31.39) was calculated after successful QC and automatic sort calibration (32,400 Hz) following the manufacturer's recommended procedure. PE CaliBRITE^TM^ beads (target) were sorted using Purity 1‐drop mode (Sort Precision Level = Purify, Drop envelope = 1), with a reported efficiency of 97.86%. (**D**) Three sequential replica CSC FCS files were concatenated in FlowJo, and regions with varying total events created in an ungated time histogram were exported as FCS files. The original analysis in FlowJo shown in **A‐C** was applied to the re‐imported CSC FCS segments and *Rmax* was calculated using Eq. 1 based on the number of target and non‐target events in the CSC and the original sample. (**E**) *Rmax* precision is affected by low count error, with a gradual decrease in spreading around the average value (94.81%) with the increase in gated *C_t_* event number.

Poisson error due to the acquisition of a small number of target and non‐target events during the analysis of the CSC fraction is another important source of error in the calculation of *Rmax*. One peculiarity of *Rmax* is that as it approaches 100% asymptotically, the number of targets collected in the CSC approaches 0 asymptotically. This has the effect of increasing the counting uncertainty for the CSC targets. This means that as *Rmax* approaches 100%, there will be more and more uncertainty in the estimation of the true *Rmax* value. The practical implication for a user is not that bleak, as the maximal *Rmax* can be inferred. First, the user must ensure that the steps in the DCD scan are far enough apart that there are enough target particles to count in the CSC as it approaches the *Rmax* maximum. Second, as a rule of thumb based on Poisson statistics, the variance *σ* ^2^ in *Rmax* values will be proportional to the number of target events being analyzed in the CSC. We have found that measuring 1000 target particles in the CSC gave good reproducibility, but collecting 1000 particles may not be feasible at *Rmax* values >95%. However, with large enough scan steps, collecting 1000 targets at the two flanking DCD delays around the maximum *Rmax* will be possible, allowing interpolation of the maximum DCD. At normal event rates, we found that a step‐size increment in DCD of ∼1/10th of a drop around the instrument‐calculated DCD gave sufficient target counts in the CSC. Obviously, for statistical accuracy, one would need to take many CSC fractions when *Rmax* values are close to 100%. For robustness, we recommend performing a minimum of three replica runs per condition in all *Rmax* calculations. It is possible to use either the mean or median values when calculating an average or median *Rmax*. In this protocol, we have adopted the mean. Please note that there is little difference between using median or mean, as the number of replicates for calculating *Rmax* is small, but being consistent in this choice is crucial. If enough CSC particles are collected, it is possible to evaluate *Rmax* using a single CSC sample collected during a sort. Figure 12 shows the error in *Rmax* for different values of acquired target events in the CSC. Capturing 400 target events reduced the *Rmax* error to <3%, which may be acceptable for the assay. In cases of very high *Rmax* values, where CSC target particles will be scarce, it may be necessary to take multiple samples to increase the accuracy of the mean *Rmax* value, or to increase the CSC volume collected per sample to take the target counts away from Poisson influence. The *Rmax* at this point will be >98% and so it may be worth the effort of collecting and processing multiple CSC to calculate *Rmax* accurately.

**Figure 13:**
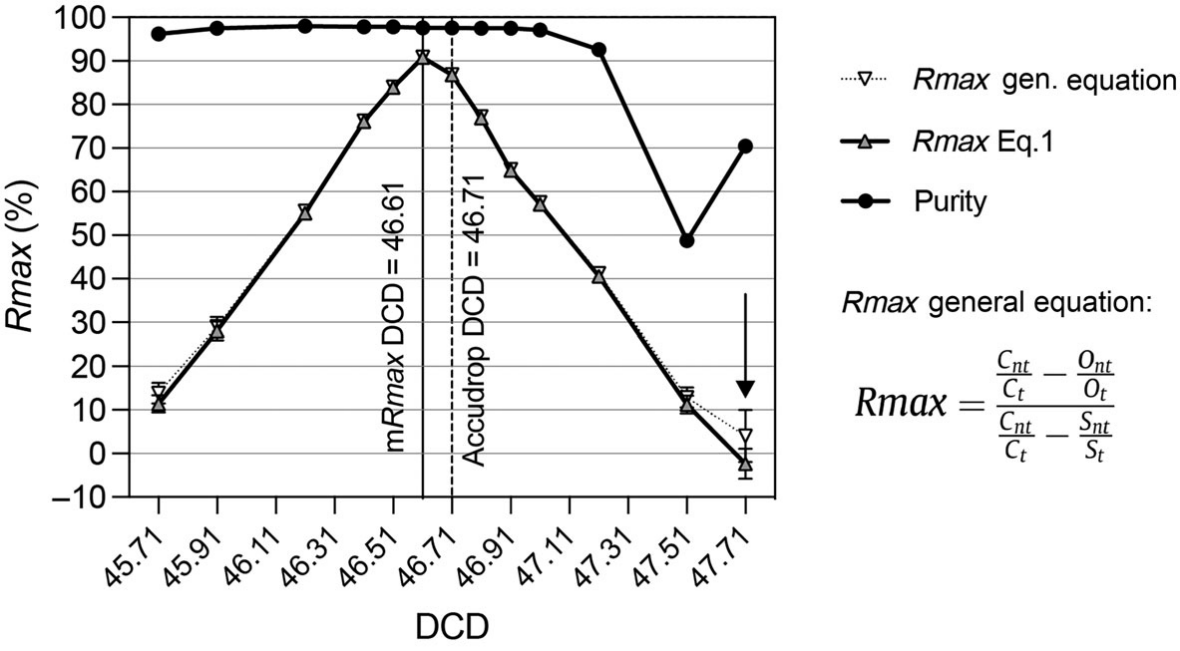
Purity and *Rmax* DCD scanning in Becton Dickinson Aria IIu sorts of BioLegend Veri‐Cells™ lymphocytes. Independent sorts were run at DCD values from 45.71 to 47.71, around the Accudrop^TM^‐ defined optimum. Original sample, sort fractions, and triplicate CSC fractions were acquired in a BD FACSCanto II analyzer, and *Rmax* was calculated (mean and error shown) based on Eq. 1 and the *Rmax* general equation (Riddell et al., 2015), which includes the non‐target to target ratio in the sort fraction. Optimum recovery of lymphocytes (*mRmax* = 90.9%) was achieved at a DCD of 46.61, very close to the Accudrop^TM^‐ defined DCD value (Accudrop^TM^ DCD = 46.71, *Rmax* = 86.9%). A close match between Eq. 1 and the *Rmax* general equation results was achieved for sort purities >90%. Note that for DCD values farthest from the optimum (∼1 drop equivalent away from the optimum DCD), *Rmax* shows minimum values and can include negative results, exhibits a large error, and shows a mismatch between values across *Rmax* equations. For these grossly suboptimal DCD values, the timing for the jet charge is either too early or too late (DCD = 47.71, arrow), with sorters deflecting leading or trailing drops surrounding those carrying the sort‐classified target. Under the typically low droplet occupancy used during sort runs, the deflected stream for these suboptimal DCD values will consist mostly of empty droplets, with very few events being sorted. Sort fraction “purities'' will resemble original target frequencies (∼66.8%, in this example). An increase in *Rmax* error and negative *Rmax* values due to Poisson low counts is expected at these suboptimal DCDs, based on Figure 12.

Follow the manufacturer‐recommended best practices for sorter setup and DCD calculation before assessing recovery with *Rmax*. To assess the dependency of the *Rmax* on DCD values for a given particle and sorter configuration, we suggest evaluating triplicate runs for 13 DCD step points, 6 on either side of the recommended DCD. Take the *Rmax* mean values of each triplicate and plot the results. A simple Excel graph is sufficient. It is not crucial to assess the purity of the sorted fractions in these experiments, as this metric is rarely affected across a wide range of DCD values around the optimum. Typical purity results from *Rmax* versus DCD scanning experiments have been included in Figures 8, 9, 11, and 13 for completeness. Optimum DCD, in terms of recovery for the particles under study, is informed by the maximum *Rmax* value observed in the DCD scanning plots. This optimum DCD value can be used for optimum sort recovery. There are circumstances where the calculated *Rmax* can differ from the manufacturer's DCD determination, as illustrated in Figures 9, 11, and 13. Notice, in Figures 11 and 13, examples of negative *Rmax* values. Negative *Rmax* values are indicative of the DCD not being timed to the last attached drop: that is, they indicate that charging was applied to the jet either too early or too late. In this scenario, most deflected drops will have not contain a particle, and those that do will contain random particle(s). In this scenario of *Rmax* analysis based on low (stochastic) particle counts, the ratio of CSC targets to non‐targets could differ from that observed in the original sample. Negative *Rmax* results will be possible here, for instance if the original sample contains a 1:1 ratio of target to non‐target particles, but the *C_t_*/*C_nt_* ratio is >1 (see Eq. 1 and Fig. 13). The negative value is corrected when we account for the sorted sample target *S_t_* and non‐target *S_nt_*, by applying the general equation for *Rmax* given in Figure 13 (Riddell et al., 2015).

As far as we are aware, there are no commercial sorters on the market that can currently set different DCD times for different populations. We have shown that in Veri‐cells™ the monocytes have a different DCD time than lymphocytes, but the resultant *Rmax* difference between the DCDs is small (Fig. 9). If both populations are to be sorted, then an optimal DCD value can be chosen between the optimum DCD observed for each population. In most cases this is usually not a problem because the difference is small. This strategy can be extended to three‐way or higher sorting setups.

A summary of the troubleshooting of issues potentially arising during the *Rmax* protocols can be found in Table 1.

**Table 1:** Troubleshooting Guide for Evaluation of Sort Recovery with *Rmax*

**Problem**	**Possible cause**	**Solution**
Bead doublets are >1%	Poor mixing of the beads	Sonicate the bead mix for 5 min and re‐acquire.
Doublets are still too high after sonication	Can be caused by old beads aggregating	Replace with a fresh batch of beads or sort total singlets into a tube and use these as the original sample.
*Rmax* values are low (*Rmax* << reported efficiency)	Malfunction of the sort electronics will give a consistent low *Rmax*; this may be due to defective sort classification in the software or electronics or malfunction in the charging of the jet or the deflection plates	Contact the instrument manufacturer for support.
	Grossly inadequate value of the DCD leading to loss in recovery using Basic Protocol 1	Repeat the sort setup and calibration following manufacturer's recommended procedures. Check for fluidic stability and consistency in BOP position before recalculating the DCD. You can briefly confirm the accuracy of the DCD by sorting a given number of bright fluorescent beads on a slide and confirming numbers under a microscope. Follow Basic Protocol 2 to scan DCD values using *Rmax* and to define the DCD optimum leading to *mRmax*.
	Inadequate sort logic excluding target events	Adjust sort gates to comprehensively include all target events in scatter, singlets, and fluorescence bivariate plots.
	Inconsistent fluorescence and scatter resolution in the analysis of the original sample and the CSC	Confirm matching sensitivity between the cell sorter used in the acquisition and sort of the original sample and the cell sorter (or analyzer) used in the analysis of the CSC fraction. If the sort logic was based on scatter parameters, excessive increase in sample differential pressure during the acquisition of the CSC could lead to changes in scatter profiles and compromise subset resolution. If the sort logic used was based on poorly expressed tertiary markers, we recommend using the same instrument for sorting and CSC analysis.
	Contamination of the CSC with the sort deflections	Check the quality of the sort deflections, their angular precision and accuracy, and the absence of fanning or “raining.” These could be evidence of fluidics instability or interactions between the cells and the droplet generation process. Increase the sort deflection angle to be far away from the CSC, to avoid cross contamination. While collecting the CSC, illuminate the center stream and sort deflections with a light torch and carefully position the CSC collection tube to avoid touching the sort deflections.
	Fluidics instability, turbulence, and changes in BOP position along the sort	The issue could be caused by inappropriate instrument startup and setup, poor fitting, and damage of fluidics components (nozzle, connectors, and switch valves), air bubbles trapped in the fluidic lines, the poor quality of the sample or the nature of the cells (size, propensity to aggregate). Check fluidics components for malfunction, or leakage. Repeat the instrument start up and setup, detectors QC, and sort calibration following manufacturer recommended procedures. Confirm fluidic stability and consistency in BOP position, before calculating the DCD following the manufacturer recommended procedures. Filter the samples to remove large aggregates. Improve the sample preparation and buffer conditions to minimize cell death and clumps. User Basic Protocol 1 to assess improvements in recovery following modifications to the sample preparation procedure.
Sort efficiency does not increase to near 100%	Excessive concentration of the bead mix	Stop the sort, remove the sample tube, and dilute the bead mix.
*Rmax* values are inconsistent among replica	Possible contamination of the CSC with the sort deflections.	Increase the sort drop deflection angle to be far away from the CSC and avoid contamination. Carefully position the CSC collection tube under the center stream to avoid touching the sort deflections.
	Insufficient BSA in the collection tubes	Ensure that there is a final concentration of 1% BSA.
	Low counts of CSC recorded; *Rmax* calculation is critically affected by Poisson counting errors and the acquisition of low counts for the CSC target events, representing, under normal sort conditions, the rarest subset in the CSC	When recording CSC fractions, set up the acquisition limit on the rarest events (target gate). If necessary, increase the volume of CSC fraction collected. To speed up the acquisition of CSC events, you can spin down the fractions, remove most of the supernatant, and resuspend particles in a smaller volume. Alternatively, you can safely record all CSC tubes on maximum flow rate for expedient process (i.e., flow rate 11 on a BD FACSAria^™^). As you do so, keep in mind that you may need to adjust the FSC × SSC gate to include the bead population, as the beads may scatter slightly lower with the widening of the core due to high differential pressure. Typically, no significant change is observed on the spreading of bright fluorescence at such high flow rates.
	Changes in total triggering events rate in replica sorts with efficiencies <100%	Ensure that the total events per second plateaus and stabilizes upon starting (or resuming) the sort and before collecting the CSC fractions. To guarantee a homogeneous cell suspension and consistent flow rates, enable automatic stirring or mixing of the sample during the sort. If that feature is not available, take note and monitor total events per second and reported sort efficiencies across replica sorts for *Rmax*. If the values change over time, stop the sort and vortex the sample before re‐acquiring it. If necessary, finely adjust the sample differential pressure to reach consistent total triggering events rate and reported sort efficiency.

### Understanding Results

*Rmax* should be used as part of the instrument calibration process, run after determining the drop delay. Using the same beads, one can record day‐on‐day *Rmax* results, much like a Levy‐Jennings plot. Any differences in the *Rmax* result will flag the instrument or operators’ contribution to the recovery. Any change in bead lots should be noted, and if necessary a new baseline should be created.

Basic Protocol 1 evaluates *Rmax* using beads as “ideal” particles and provides a single measure of the sort recovery for a given instrument configuration. When sorting bead mixes with Purity 1‐drop mode in a perfectly operating instrument, *Rmax* values are expected to be very close to the reported sort efficiency (see Fig. 12, calculated *Rmax* = 94.8% vs. sort efficiency = 97.8%). A small loss of recovery compared to reported efficiencies in 1‐drop Purity mode is expected, owing to particles’ behavior in the jet and not to instrument malfunction: particles close to the drop boundaries may migrate into the flanking drops. This is a natural outcome of the uncertainty in particle position within the BOP drop. *Rmax* will reflect this loss and provide a better measure of recovery than the reported sort efficiency.

A critical issue leading to loss during recovery is inaccuracy of the DCD calculated during sort setup. Note that using purity alone to define the sort performance metric is folly. Figures 8, 9, 11, and 13 show results obtained when using Basic Protocol 2 to scan through the DCD values using a Purity 1‐drop sort mode. Because in this mode the electronics keep the sort pure, the purity data in almost all cases is near 100% for most of the DCD values evaluated. In contrast, recovery in all these examples shows a strict dependency on the DCD, with a sharp reduction in values when moving away from the *mRmax* DCD optimum. Using recovery as the main metric of sort performance, Basic Protocol 2 can be used to assess whether losses in sort target recovery and deviations from reported efficiencies observed via Basic Protocol 1 are due to an inaccurate DCD setting. Subsequently, Basic Protocol 2 is used to identify the optimum DCD. This provides the maximum recovery values for a given set of conditions and sample preparation. Choosing a 1‐drop envelope is critical in these evaluations to find the optimum DCD timing. Figure 10 shows a flat *Rmax* curve obtained when using a Purity 1‐2 sort envelope. This relative insensitivity of the 1‐2‐drop envelope to imprecise DCD values preserves recovery, but the trade‐off is that one must use a slower sample event rate to maintain acceptable abort rates.

As discussed, *Rmax* provides a better estimate of recovery than theoretical predictions based on Poisson/binomial distributions or instrument efficiency reports. The ratiometric nature of *Rmax* brings greater accuracy to the recovery calculation as compared to absolute count methods that involve sampling the sorted fraction. However, both precision and accuracy of the metric are affected by several factors: changes in total event rate when *Rmax* is calculated while running sorts at <100% efficiency, CSC sampling cross‐contamination from sort deflections, and low‐number Poisson counting errors. CSC must be collected while sorting at a consistent event rate when reported sort efficiency is <100%. Here, replica sampling of the CSC will be necessary to identify and discard potential outliers (Fig. 11) and to compensate for low target counts (Fig. 12). Guaranteeing the acquisition of enough events for target and non‐target particles during the analysis of original and replica CSC samples will minimize errors derived from stochastic low counts (Fig. 12D). This is observed when collecting the CSC near the optimum DCD, at which values of *Rmax* approach its maximum and the number of target events in the CSC approaches 0, confounding *Rmax* precision and accuracy. All is not lost, however, as this effect can be used as a strong indicator of the *mRmax*, and an increased number of replica CSC samples may yet improve the accuracy of these results (Fig. 12). Negative values of *Rmax* can be computed and represent sampling errors caused by the DCD deflecting a drop either too early or too late for the target particle's arrival at the BOP. The CSC is then enriched with targets, causing the negative value in the *Rmax* calculation. Interestingly, these negative *Rmax* values mark the droplet boundaries of the DCD timing.

*Rmax* determination via Basic Protocol 1 or 2 can be used to set performance baselines upon the installation of a new cell sorter, for monitoring performance, and for the early identification of problems, such as whether the instrument may not be in perfect condition or the physical properties of particles lead to flow deviating from Poissonian behavior. We recommend that differences between reference and daily *Rmax* values of >5% be taken as an indication of problems with the instrument. Basic Protocol 2 is also used to assess sorter performance and set recovery baselines after any major maintenance or repair procedures. Most commonly, changes in *Rmax* could reflect improper DCD definition, which can be confirmed via DCD scanning with Basic Protocol 2. Other instrument issues leading to poor *Rmax* include fluidic instability, improper sort setup, and sort electronics subsystem failure. These can be remedied by following the instrument's troubleshooting guide or through a field service engineer visit, taking *Rmax* measurements to check for suitable improvements. In instrument sort performance monitoring, Basic Protocol 2 can be used to evaluate and compare similar instruments from the same manufacturer, when the *mRmax* deviation should be no more than 5%.

Sorting cells poses its own challenges. Cells tend to flow in a non‐Poissonian way and are generally heterogenous compared to beads. The *mRmax* will strongly depend on the cell type and the quality of the sample preparation. The use of *Rmax* when sorting cells falls into two categories: sample optimization and sort monitoring. Using Basic Protocol 1, when developing and optimizing sample preparation protocols for cell sorting, *Rmax* can be used to measure the impact of different strategies on recovery at each stage of the assay development cycle. To expound, a poor *Rmax* is usually a result of cell death, problems with aggregate exclusion, or cell loss from adherence to tube walls. As shown in Figure 9, improvements to the sample preparation may be made to increase the *Rmax v*alues and lead to successful outcomes. In monitoring the cell sorting process, repeated *Rmax* sampling and averaging will help improve the estimate of recovery. During a sort, measuring *Rmax* intermittently monitors the sort progression and can be used to predict the total sort number at the end of the allotted time.

Finally, if a certain number of target cells is required, Eq. 4 in Basic Protocol 3 can be used to adjust the sorting time to reach the desired number of events.

### Time Considerations

Basic Protocol 1—Evaluating sorter setup with *Rmax*: Once reagents and supplies are prepared and cell sorter startup and setup are done, the *Rmax* protocol should take 35 min to complete: 15 min for sorting and collection, 5 min for cleaning, and 15 min for recording triplicate tubes. This estimate assumes that all templates for acquisition software are defined and in place ahead of time.

Basic Protocol 2—Finding the maximum *Rmax*: scanning over the DCD: Once reagents and supplies are prepared and cell sorter startup and setup are done, this *Rmax* protocol should take 4 hr to complete. This estimate assumes that all templates for acquisition software are defined and in place ahead of time.

Alternate Protocol—Finding the maximum *Rmax* for cells: scanning over the drop delay: Once cells, reagents, and supplies are prepared and cell sorter startup and setup are done, the *Rmax* protocol should take 3.5 hr to complete, 30 min for sorting and collection, and 5 min for cleaning. A further 140 min is needed for recording triplicate tubes, along with a total of 35 min of cleaning between each triplicate.

Basic Protocol 3—Estimating sorted cell number from *Rmax*: Once cells, reagents, and supplies are prepared and cell sorter startup and setup are completed, the *Rmax* protocol should take the same time as the sort if using a separate analyzer. Otherwise, if you are using the sorter to analyze the CSC fractions, it should take the same amount of time as Basic Protocol 1 (35 min).

### Author Contributions

**Alexis Perez‐Gonzalez**: Conceptualization; data curation; formal analysis; investigation; methodology; resources; supervision; validation; visualization; writing—original draft; writing—review and editing. **Telma Lopes**: Data curation; formal analysis; investigation; methodology; resources; writing—original draft; writing—review and editing. **Lola Martinez**: Conceptualization; data curation; formal analysis; investigation; methodology; resources; writing—original draft; writing—review and editing. **Claudia Bispo**: Data curation; formal analysis; methodology; resources; validation; writing—original draft; writing—review and editing. **Rui Gardner**: Conceptualization; data curation; formal analysis; investigation; methodology; resources; software; supervision; validation; visualization; writing—original draft; writing—review and editing. **Andy Riddell**: Conceptualization; data curation; formal analysis; funding acquisition; investigation; methodology; project administration; resources; software; supervision; validation; visualization; writing—original draft; writing—review and editing.

### Conflict of Interest

The authors declare no conflict of interest.

## Supporting information




*Supplemental Table 1 CSC collection strategy by instrument*.

## Data Availability

The data, tools, and material (or their source) that support the protocol are available from the corresponding author upon reasonable request.

## References

[cpz1986-bib-0001] Beckman Coulter (2017). MoFlo Astrios, MoFlo AstriosEQ, and MoFlo Astrios EQS Instructions for use. Chapter 8, p. 38. Retrieved from https://www.beckman.com

[cpz1986-bib-0002] Beckman Coulter (2022). CytoFLEX SRT Instructions for use. Chapter 5, p. 5.2. Retrieved from https://www.beckman.com

[cpz1986-bib-0003] Bio‐Rad (2013). S3 Cell Sorter Instruction Manual, 4.4.4 Drop Delay Determination Catalog #145‐1001, #145‐1002, pp. 34–35.

[cpz1986-bib-0004] Brando, B. , Göhde, W. Jr. , Scarpati, B. , & D'Avanzo, G. (2001). European Working Group on Clinical Cell Analysis. The “vanishing counting bead” phenomenon: Effect on absolute CD34^+^ cell counting in phosphate‐buffered saline‐diluted leukapheresis samples. Cytometry, 43(2), 154–160. 10.1002/1097-0320 11169581

[cpz1986-bib-0005] Dacie, S. J. V. , & Lewis, S. M. (1984). Practical haematology (6th ed.) pp. 26–27. Churchill Livingtone.

[cpz1986-bib-0006] Ibrahim, S. F. , & van den Engh, G. (2007). Flow cytometry and cell sorting. Advances in Biochemical Engineering/Biotechnology, 106, 19–39. 10.1007/10_2007_073 17728993

[cpz1986-bib-0007] Kirkman‐Brown, J. , & Björndahl, L. (2009). Evaluation of a disposable plastic Neubauer counting chamber for semen analysis. Fertility and Sterility, 91(2), 627–631. 18439603 10.1016/j.fertnstert.2007.11.076

[cpz1986-bib-0008] Lapping, T. R. J. , Lamont, A. , & Nelson, M. G. (1972). An evaluation of the Celloscope 401 electronic blood cell counter. Journal of Clinical Pathology, 25(6), 539–542. 4625438 10.1136/jcp.25.6.539 PMC477376

[cpz1986-bib-0009] Lazebnik, Y. A. , Poletaev, A. I. , & Zenin, V. V. (1992). Drop‐delay measurement using enzyme‐coated particles. Cytometry, 13(6), 649–652. 10.1002/cyto.990130614 1451596

[cpz1986-bib-0010] Lindmo, T. , & Fundingsrud, K. (1981). Measurements of the distribution of time intervals between cell passages in flow cytometry as a method for the evaluation of sample preparation procedures. Cytometry, 2(3), 151–154. 10.1002/cyto.990020303 6170497

[cpz1986-bib-0011] Nicholson, J. K. , Stein, D. , Mui, T. , Mack, R. , Hubbard, M. , & Denny, T. (1997). Evaluation of a method for counting absolute numbers of cells with a flow cytometer. Clinical and Diagnostic Laboratory Immunology, 4(3), 309–313. 9144369 10.1128/cdli.4.3.309-313.1997 PMC170524

[cpz1986-bib-0012] Norton, P. O. (2002). Apparatus and method for verifying drop delay in a flow cytometer. US Patent Number US 6,372,506 B1.

[cpz1986-bib-0013] Pegg, D. E. , & Antcliff, A. C. (1965). An evaluation of the Vickers Instruments J12 cell counter. Journal of Clinical Pathology, 18(4), 472–478. 14318703 10.1136/jcp.18.4.472 PMC472983

[cpz1986-bib-0014] Petersen, T. W. , & van den Engh, G. (2003). Stability of the breakoff point in a high‐speed cell sorter. Cytometry Part A: The Journal of the International Society for Analytical Cytology, 56(2), 63–70. 10.1002/cyto.a.10090 14608633

[cpz1986-bib-0015] Riddell, A. , Gardner, R. , Perez‐Gonzalez, A. , Lopes, T. , & Martinez, L. (2015). *Rmax*: A systematic approach to evaluate instrument sort performance using center stream catch. Methods, 82, 64–73. 10.1016/j.ymeth.2015.02.017 25747337 PMC4503806

[cpz1986-bib-0016] Shapiro, H. M. (2004). Practical flow cytometry (4th ed., p. 267). John Wiley & Sons.

[cpz1986-bib-0017] Stovel, R. T. (1977). The influence of particles on jet breakoff. Journal of Histochemistry & Cytochemistry, 25(7), 813–820. 10.1177/25.7.894007 894007

[cpz1986-bib-0018] van Dilla, M. A. , Dean, P. N. , Laerum, O. D. , & Melamed, M. R. (Eds.) (1985). Flow cytometry instrumentation and data analysis p. 288. Academic Press.

[cpz1986-bib-0019] Vitelli, M. , Budman, H. , Pritzker, M. , & Tamer, M. (2021). Applications of flow cytometry sorting in the pharmaceutical industry: A review. Biotechnology Progress, 37, e3146. 10.1002/btpr.3146 33749147

[cpz1986-bib-0020] Watson, J. V. (1991). Introduction to flow cytometry p. 443. Cambridge University Press.

