# Supplementary material for: Evaluation of Sort Recovery via Rmax
Source: Curr Protoc. 2024 Feb 16;4(2):e986. doi: 10.1002/cpz1.986 (PMC13014063; doi:10.1002/cpz1.986)
Supplement: Supplementary file 1 — Supplemental Table 1 CSC collection strategy by instrument. [file CPZ1-4-0-s001.pdf]

Supplementary Material

Evaluation of sort recovery via Rmax

Alexis Perez-Gonzalez[1,2], Telma Lopes[3], Lola Martinez[4], Claudia Bispo[5], Rui Gardner[6] and Andy Riddell[7]

1 Department of Microbiology and Immunology, The University of Melbourne, at The Peter Doherty Institute of Infection and Immunity, Parkville, Vic, 3010, Australia.

2 Melbourne Cytometry Platform, The University of Melbourne, Parkville, Vic, 3010, Australia

3 Roche Pharma Research and Early Development (pRED), F. Hoffmann-La Roche AG, Basel. Switzerland.

4 Flow Cytometry Core Unit. Spanish National Cancer Research Center (CNIO). C/ Melchor Fernández Almagro, 3 28029. Madrid. Spain.

5 Flow Cytometry Core Facility, AbbVie Biotherapeutics Inc., 1000 Gateway Blvd, South San Francisco, CA 94080. USA.

6 Flow Cytometry Core Facility, Memorial Sloan Kettering Cancer Center, 417 East 68th Street, New York, NY 10065. USA.

7 Flow Cytometry Science and Technology Platform, The Francis Crick Institute, Midland Road London NW1 1AT UK, +44 20379 65199, andy.riddell@crick.ac.uk

WARNING: A risk assessment **MUST** be performed before proceeding. You **MUST** follow all local safety rules including local laser safety rules when collecting the CSC. Some instruments may require the laser interlocks or the sort chamber interlock to be bypassed in order to collect CSC fractions. There is a potential for electrocution from the deflection plates and proper steps outlined by your risk assessment and local safety rules **MUST** be followed. If unsure, **DO NOT PROCEED** and contact the instrument manufacturer for advice. **You do this at your own risk, no liability is taken nor implied by the authors or the journal.**

SUPPLEMENTAL TABLE 1 CSC collection strategy by instrument

**Instrument model (manufacturer)**	**Collecting the Center Stream**	**Override Laser Shutter / other?**
MoFlo ASTRIOS (Beckman Coulter)	While running the sort, adjust the center stream charge (waste) to deflect it into the CSC collection tube, placed opposite to the sort collection tube. 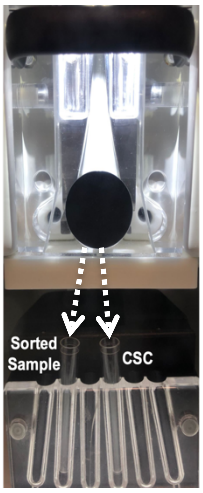	Not needed

SUPPLEMENTAL TABLE 1 CSC collection strategy by instrument, *continued*

<table>
<tr><th>Instrument model (manufacturer)</th><th>Collecting the Center Stream</th><th>Override Laser Shutter / other?</th></tr>
<tr><td>BD FACS ARIA (Becton Dickinson)</td><td>Place the CSC collection tube under the center steam while the sort is in progress.
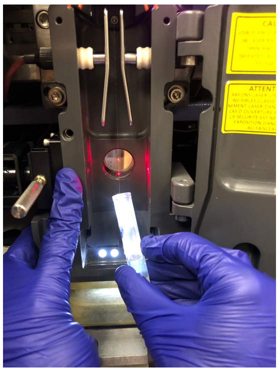</td><td>WARNING: POSSIBLE EXPOSURE TO LASER RADIATION. You MUST follow Local Laser Safety Rules.
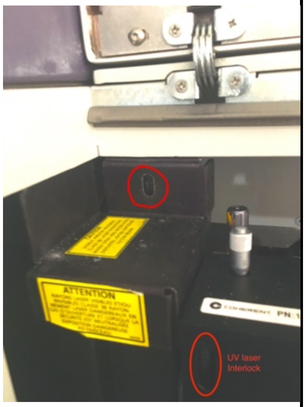

Open the sort chamber lid. There are 2 interlocks depending on the instrument laser configuration.
If you don’t have the U.V. laser then insert a pipette tip or an Allen key to wedge open the defeat.
If the sorter has a UV laser then you will need to defeat the UV interlock the same way. The UV interlock is found under the top interlock. Push a pipette tip or Allen key into the hole to override laser interlock.</td></tr>
<tr><td>BD FACS ARIA Fusion (Becton Dickinson)</td><td>Place the CSC collection tube under the center steam while the sort is in progress.
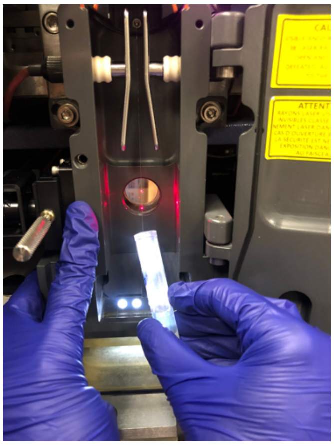</td><td>WARNING POSSIBLE EXPOSURE TO LASER RADIATION. You MUST follow Local Laser Safety Rules.
Open the lid of the sort chamber. The interlock defeat is found at the top right-hand side of the chamber. Pull the button to the left to defeat the interlock.
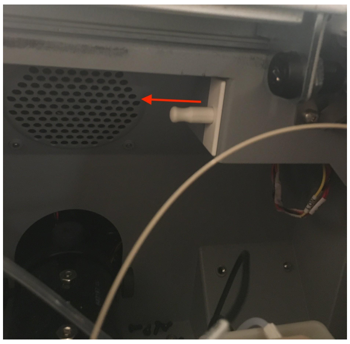</td></tr>
</table>

SUPPLEMENTAL TABLE 1 CSC collection strategy by instrument, *continued*

**Instrument model (manufacturer)**	**Collecting the Center Stream**	**Override Laser Shutter / other?**
Influx (Becton Dickinson)	Place the CSC collection tube in a suitable rack and under the center steam. While the sort is in progress, push in the waste stream catching tube to let the stream into the CSC collection tube. 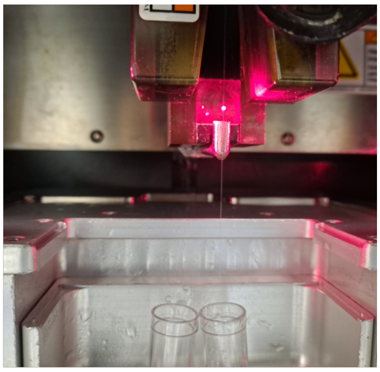	Not needed
MoFlo Legacy or XDP (Beckman Coulter)	Adjust the center stream charge to deflect the waste stream in the opposite direction to your sort stream. 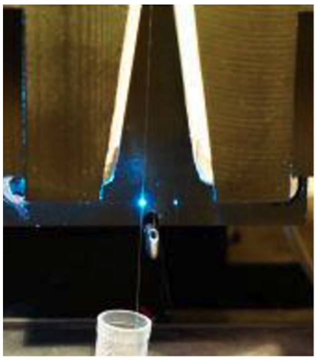	Not needed
BioRad S3 (Propel Avalon)	Collect CSC directly. *Note: The instrument defaults to a 1-2 drop envelope. It is advised to speak to your service engineer to set the sort drops to 1 drop deflection.*	Sort Chamber interlock override. This is found under the top cover above the sheath and waste tanks. Wire a Molex 6 pin male plug as below. 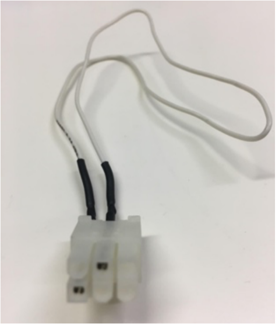

SUPPLEMENTAL TABLE 1 CSC collection strategy by instrument, *continued*

**Instrument model (manufacturer)**	**Collecting the Center Stream**	**Override Laser Shutter / other**
BioRad S3 (Propel Avalon). *Continued*	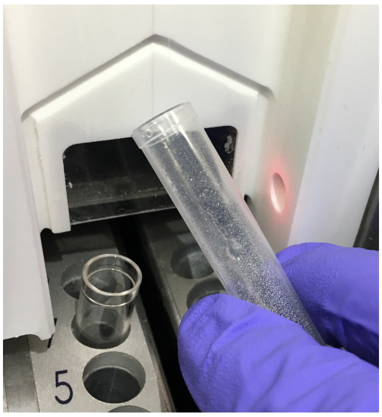	Insert this into the SRT lead to defeat the interlock on the sort chamber. 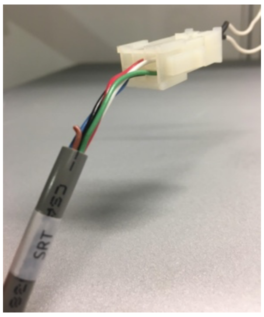
Sony SH800 or MA900	Collect CSC directly. Place the CSC collection tube under the center steam while the sort is in progress	Hold door latch on the left to defeat the the sort chamber interlock. 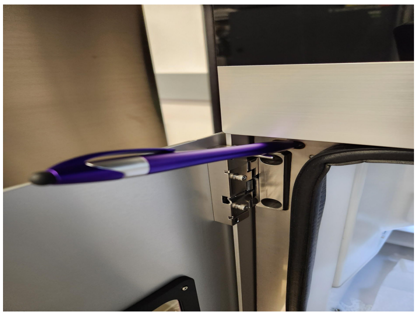
CytoFLEX SRT (Beckman Coulter)	Collect CSC directly. Place the CSC collection tube under the center steam while the sort is in progress. 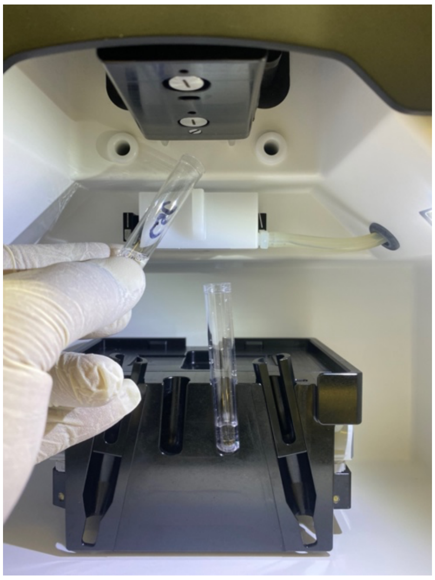	Not needed
